# Structures of the NDP-pyranose mutase belonging to glycosyltransferase family 75 reveal residues important for Mn^2+^ coordination and substrate binding

**DOI:** 10.1016/j.jbc.2023.102903

**Published:** 2023-01-13

**Authors:** Xueqing Du, Xuan Chu, Ning Liu, Xiaoyu Jia, Hui Peng, Yazhong Xiao, Lin Liu, Haizhu Yu, Fudong Li, Chao He

**Affiliations:** 1 Anhui Key Laboratory of Modern Biomanufacturing and School of Life Sciences, Anhui University, Hefei, Anhui, China; 2 Department of Chemistry and Centre for Atomic Engineering of Advanced Materials, Anhui Province Key Laboratory of Chemistry for Inorganic/Organic Hybrid Functionalized Materials, Anhui University, Hefei, China; 3 MOE Key Laboratory for Cellular Dynamics, School of Life Sciences, Division of Life Sciences and Medicine, University of Science and Technology of China, Hefei, China

**Keywords:** GT75, NDP-furanose mutase, glycosyltransferase, crystal structure, catalytic mechanism, ASU, asymmetric unit, FAD, flavin adenine dinucleotide, Fucf, GDP-L-fucofuranose, Fucp, GDP-L-fucopyranose, GT75, glycosyltransferase family 75, HILIC, hydrophilic interaction chromatography, ITC, isothermal titration calorimetry, L-Arap, UDP-L-arabinopyranose, L-Galf, GDP-L-galactofuranose, L-Galp, GDP-L-galactopyranose, PDB, Protein Data Bank, RGP, reversibly glycosylated polypeptide, SLC, static light scattering, TEV, tobacco etch virus, TOF-MS, time-of-flight mass spectrometry, UAM, UDP-L-arabinopyranose mutase, UDP-D-Galp, UDP-D-galactopyranose, UDP-D-Glcp, UDP-D-glucopyranose, UGM, UDP-D-galactopyranose mutase, UPLC, ultrahigh-performance liquid chromatography

## Abstract

Members of glycosyltransferase family 75 (GT75) not only reversibly catalyze the autoglycosylation of a conserved arginine residue with specific NDP-sugars but also exhibit NDP-pyranose mutase activity that reversibly converts specific NDP-pyranose to NDP-furanose. The latter activity provides valuable NDP-furanosyl donors for glycosyltransferases and requires a divalent cation as a cofactor instead of FAD used by UDP-D-galactopyranose mutase. However, details of the mechanism for NDP-pyranose mutase activity are not clear. Here we report the first crystal structures of GT75 family NDP-pyranose mutases. The novel structures of GT75 member MtdL in complex with Mn^2+^ and GDP, GDP-D-glucopyranose, GDP-L-fucopyranose, GDP-L-fucofuranose, respectively, combined with site-directed mutagenesis studies, reveal key residues involved in Mn^2+^ coordination, substrate binding, and catalytic reactions. We also provide a possible catalytic mechanism for this unique type of NDP-pyranose mutase. Taken together, our results highlight key elements of an enzyme family important for furanose biosynthesis.

Members of glycosyltransferase family 75 (GT75) in the carbohydrate-active enzymes database commonly possess two distinct types of enzymatic activities, a glycosyltransferase activity that autoglycosylates a conserved arginine residue by nucleoside diphosphate (NDP)-sugars and an NDP-pyranose mutase activity that interconverts NDP-pyranose and NDP-furanose. GT75 currently contains 308 entries from eukaryotes, bacteria, and archaea. MtdL and hyg20 are the only two characterized bacterial members of this family. MtdL from deep sea–derived *Marinactinospora thermotolerans* SCSIO 00652 has been discovered in the antibiotic A201A biosynthetic pathway, generating GDP-L-galactofuranose (L-Galf) from GDP-L-galactopyranose (L-Galp) (1). Hyg20 from *Streptomyces hygroscopicus* NRRL 2388 is responsible for the conversion of GDP-L-fucopyranose (Fucp) to GDP-L-fucofuranose (Fucf) in the biosynthesis of the aminoglycoside antibiotic hygromycin (2). Both MtdL and Hyg20 enzymes can catalyze interconversion between GDP-L-Fucp and GDP-L-Fucf, and interconversion between GDP-L-Galp and GDP-L-Galf *in vitro* (2).

Except for MtdL and hyg20, all other characterized members in GT75 family were from plants. Some plant members, named as UAMs, have been characterized to have UDP-L-arabinopyranose (L-Arap) mutase (UAM) activity, which converts UDP-L-Arap to UDP-L-Araf (3). Such UAM activity represents the only known route to L-Araf in plants, playing essential roles in plant cell wall integrity as L-arabinose moieties are found predominantly in the furanose form in plant cell wall components (4). Plant UAMs also belong to a small family of proteins originally identified as reversibly glycosylated polypeptides (RGPs), which can be reversibly autoglycosylated at a conserved arginine residue (corresponding to Arg158 in OsUAM1 from *Oryzae sativa*) by a variety of UDP-sugars, such as UDP-D-glucopyranose (UDP-D-Glcp), UDP-D-galactopyranose (UDP-D-Galp), and UDP-D-xylopyranose (UDP-D-Xylp) (5, 6). In contrast, this RGP activity has not been characterized in bacterial members. The conserved glycosylated arginine residue is required for mutase activity in plant members, such as OsUAM1 (7), OsUAM3 (7) and barley HvUAM1 (8). Intriguingly, some plant members displayed no UAM or RGP activity, such as Arabidopsis AtRGP5 (4), OsUAM2 (9) and HvUAM4 (8).

Despite the importance of GT75 family in furanose biosynthesis, the molecular mechanism underlying their enzymatic activities remains elusive. Their mutase activities were reminiscent of another mutase activity catalyzed by flavin adenine dinucleotide (FAD)-dependent UDP-D-galactopyranose mutases (UGMs) that interconvert UDP-D-Galp and UDP-D-Galf (10, 11). However, the GT75 family mutases do not bind FAD, and FAD is not necessary for the activity. In contrast, both mutase and autoglycosyltransferase activities of GT75 members are dependent on a divalent cation (2, 12), specifically Mn^2+^ or Mg^2+^. Meanwhile, there is a highly conserved DDD motif across GT75, which has been shown to be indispensable for UAM activity in OsUAM1 (12) and HvUAM1 (8). This motif might coordinate a Mn^2+^ ion that electrostatically stabilizes the negative charge developing on the diphosphate of the NDP-sugar substrate as shown by the characteristic DXD motif in GT-A fold glycosyltransferases (13). Therefore, the GT75 family represents a new type of NDP-pyranose mutase distinct from previously reported UGMs and must have a unique catalytic mechanism.

Here, we report a collection of MtdL structures, which, together with site-directed mutagenesis studies, provide deep insights into the catalytic mechanism of the GT75 family for the first time. We obtained crystal structures of wildtype (WT) MtdL in complex with GDP, GDP·Mn^2+^, and GDP-D-Glcp·Mn^2+^, respectively. We also obtained crystal structures of the catalytically deficient S228A mutant of MtdL in complex with GDP-L-Fucp·Mn^2+^ and GDP-L-Fucf·Mn^2+^, respectively, and the R257K mutant bound with Mn^2+^. These six structures showed that MtdL is composed of an N-terminal GT-A fold, novel core, and C-terminal helix domains. The characteristic DXD motif (Asp109-Asp110-Asp111), together with conserved His287, coordinates a Mn^2+^ ion, which stabilizes the diphosphate of the nucleotide. Moreover, isothermal titration calorimetry (ITC) analyses of MtdL-GDP binding and high-performance liquid chromatography (HPLC) analyses of reaction mixtures catalyzed by MtdL and AtRGP2, including both WT and designed mutants, combined with comparative structural analysis, revealed critical substrate binding and potential catalytic residues for this unique type of NDP-pyranose mutase. Our study also revealed a conserved hydrophobic dimerization interface in this family.

## Results

### Overall structure of MtdL

The initial structure of MtdL at 2.1-Å resolution was determined using single-wavelength anomalous diffraction phasing from cocrystals of selenomethionine (SeMet) derivative MtdL with GDP. However, the electron density of the GDP diphosphate moiety was poor (Fig. S1, *A* and *C*). Then, the complex structure of MtdL bound to Mn^2+^ and GDP at 2.1-Å resolution was obtained by cocrystallizing MtdL containing 0.5 mM MnCl_2_ with GDP, which led to a clear electron density for both the GDP diphosphate moiety and the Mn^2+^ ion at the active site (Fig. S1, *B* and *D*). Both SeMet-MtdL·GDP and MtdL·Mn^2+^·GDP structures contain two protein chains per asymmetric unit (ASU) that can be superimposed with root-mean-square deviations (RMSDs) of 0.079 A˚ and 0.081 A˚ over 308 and 353 Cα atoms, respectively.

**Figure 1:**
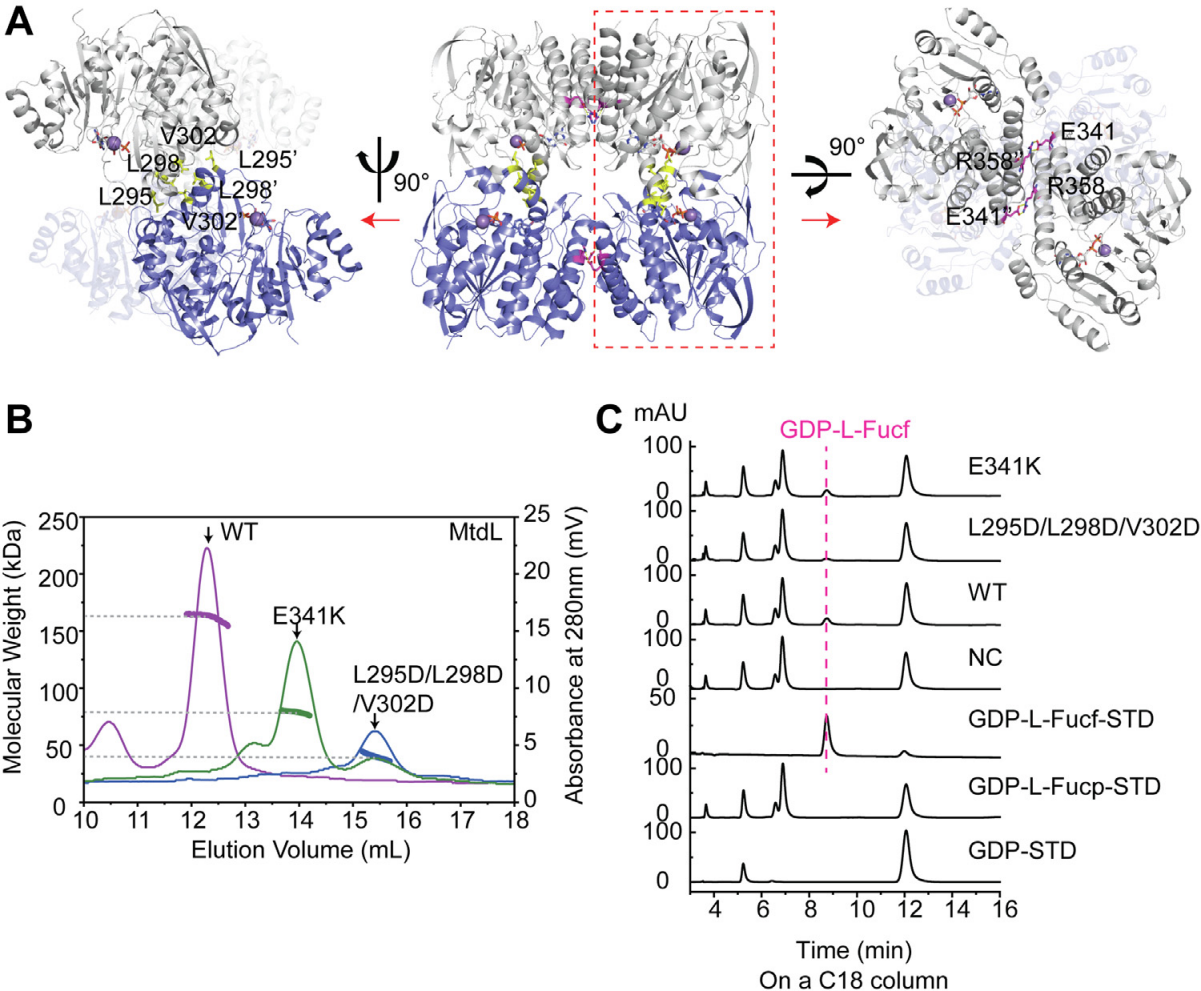
**Dimerization interfaces in tetramer formation of MtdL.***A*, in the crystal of GDP·Mn^2+^·MtdL belonging to space group *C*222_1_, one ASU highlighted by *red* dotted squares containing two chains (chain A and B colored in *gray* and *blue*, respectively) is half a tetramer. The hydrogen-bonding and hydrophobic dimerization interfaces in tetramer formation of MtdL are shown. *B*, static light scattering profiles of recombinant MtdL WT, its E341K and L295D/L298D/V302D mutants shown as *purple*, *green*, and *blue lines*, respectively. The resulting experimental molecular mass of WT MtdL is 164.8 ± 2.7 kDa; the expected molecular mass for the single chain including a His_6_ tag is 41.6 kDa, demonstrating that it forms a tetrameric structure. The resulting experimental molecular mass of the E341K mutant of MtdL is 79.95 ± 0.84 kDa, demonstrating that this mutant forms a dimeric structure. The resulting experimental molecular mass of the L295D/L298D/V302D mutant of MtdL is 42.21 ± 1.35 kDa, demonstrating that this three-point mutant becomes a monomer. *C*, HPLC analyses of the *in vitro* reaction mixtures catalyzed by MtdL WT, its E341K and L295D/L298D/V302D variants.

A tetramer as a dimer of dimers was suggested by PISA for crystal structures of both GDP·SeMet-MtdL belonging to space group *C*2 and GDP·Mn^2+^·MtdL belonging to space group *C*222_1_ (Figs. 1*A* and S2). Consistently, MtdL was discovered as a tetramer in solution through static light scattering (SLC) (Fig. 1*B*). Detailed dimerization interfaces in tetramer formation and their effects on enzymatic activity will be described later. We begin with the description of the structure of each MtdL subunit.

**Figure 2:**
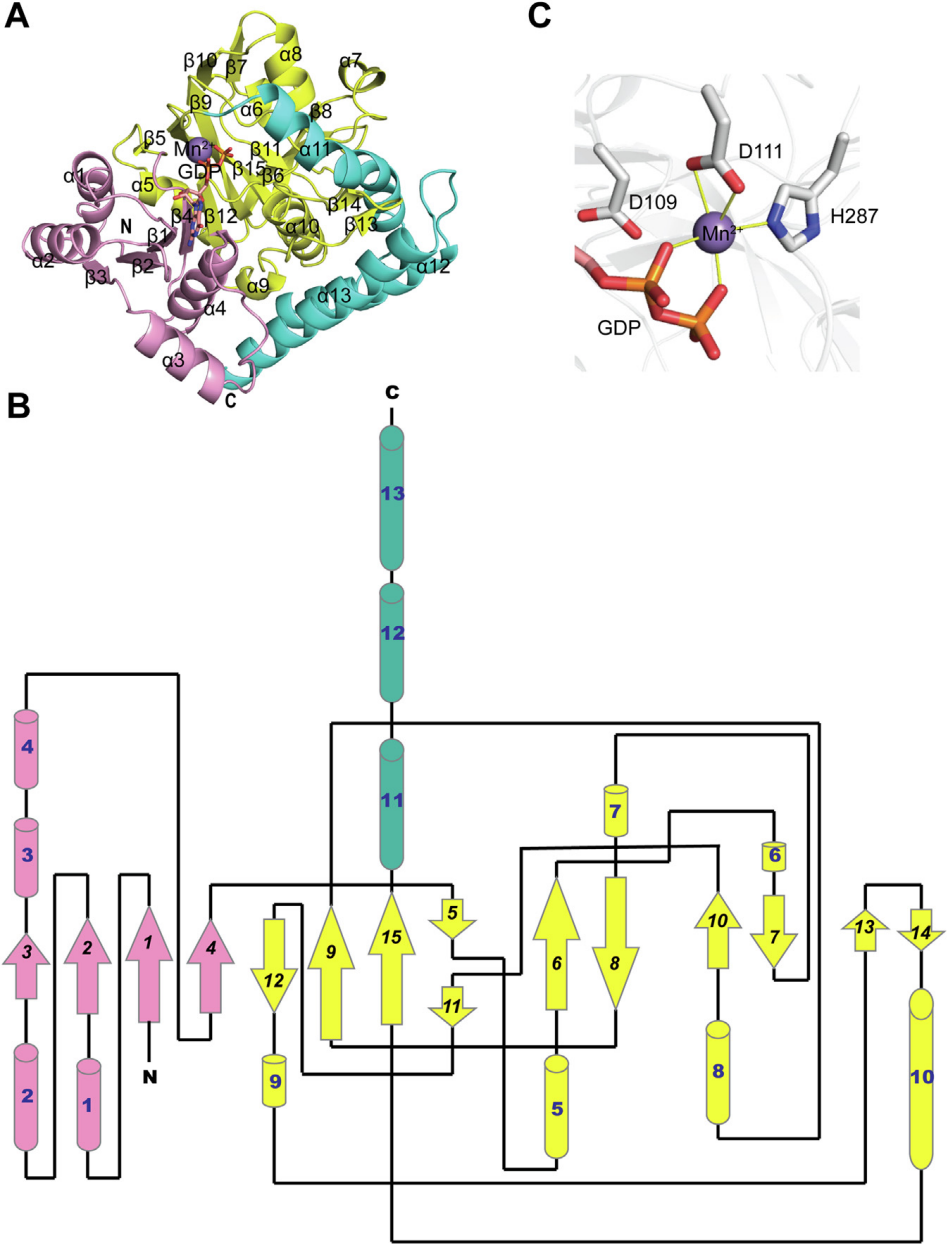
**Overall structure of each MtdL subunit.***A*, the MtdL subunit is shown in ribbon representation in complex with Mn^2+^ and GDP. The secondary structure was calculated using the Define Secondary Structure of Proteins algorithm. The N-terminal region with a Rossmann-like fold (residues 1–111) is colored *pink*. The core region (residues 112–291) is shown in *yellow.* The C-terminal helix region (residues 292–376) is shown in *green.* The GDP and Mn^2+^ ion are shown in stick and in *gray sphere*, respectively. *B*, topology diagram of MtdL. *C*, the Mn^2+^ coordination bonds are shown as *yellow full lines*.

The MtdL subunit is divided into three regions (Fig. 2*A*): the N-terminal region (residues 1–111), the core region (residues 112–291), and the C-terminal region (residues 292–376). As shown in the topology diagram of MtdL (Fig. 2*B*), the N-terminal region comprised four parallel β-strands (topology β3-β2-β1-β4) surrounded by four helices (α1 to α4) to form a Rossmann-like fold. The core region consists of 11 strands and 6 helixes. Strand β4 connects the N-terminal region with the core region of the protein by forming an antiparallel β-sheet with β12. Strands β12-β9-β15-β11 lined in the center clamped by α5 and α10. Two twisted antiparallel β-sheets (a longer one formed by β6 and β8 and a shorter one formed by β7 and β10) sit at the edges of the core region. Another antiparallel β-sheet formed by β13 and β14 protrudes into the C-terminal region, which consists of three helices (α11 to α13).

We next compared the overall structure of the MtdL single chain (residues 1–376) with other structures in the Protein Data Bank (PDB) using the DALI server and identified a number of glycosyltransferases of the GT-A fold. The GT-A fold is characterized by a Rossmann-like fold for donor nucleotide binding. The closest structural matches with a Z-score of 15.4 were UDP-galactofuranosyl transferase GlfT2 (14) (PDB code 4fix, RMSD of 3.4 Å for 228 Cα atoms, and 11% sequence identity), UDP-N-acetylglucosamine glycosyltransferase CgT (15) (PDB code 5hea, RMSD of 3.5 Å for 216 Cα atoms, and 12% sequence identity), and chondroitin synthase (16) (PDB code 2z87, RMSD of 3.8 Å for 215 Cα atoms, and 14% sequence identity). All the three structural homologues were grouped into the GT2 family with an inverting catalytic mechanism suggested to be employed by RGPs (17). Structural alignments of the MtdL single chain to the three closest structural matches by using CEalign in PyMOL revealed that only the Rossmann-like-fold region and part of the core region could be superimposed. No meaningful similarity was found for the core region and the C-terminal helix domain. These results suggested that MtdL contains unique core and helix domains and a typical GT-A-type glycosyltransferase domain and thus represents a new member of GT-A-type glycosyltransferases.

### Mn^2+^ coordination

In the Mn^2+^·GDP bound structure, the Mn^2+^-binding site (Fig. 2*C*) is formed by Asp111 at the third position of the DXD motif on the loop following β4 and a conserved histidine His287 at the end of β15. This Mn^2+^ coordination is slightly different from that observed in other GT-A glycosyltransferases, where both aspartates in the DXD motif coordinate to Mn^2+^ directly. In addition, each phosphate group of GDP coordinates to Mn^2+^ through a single oxygen atom, as shown in other GT-A enzymes.

Structural comparison of GDP-MtdL complexes in the absence and presence of Mn^2+^ indicated a major conformational change in loop A (residues 288–292), which follows the Mn^2+^ coordinating His287 on β15 (Fig. S1*E*). This flexible loop, together with the diphosphate of GDP, showed poor electron density in the GDP bound structure without Mn^2+^ (Fig. S1*A*). By contrast, loop A displayed two conformations in the Mn^2+^·GDP bound structure (Fig. S1*B*). One is distal from the diphosphate group, similar to that shown without Mn^2+^, while the other is in the vicinity of the diphosphate. The side chains of Arg289 and His292 on loop A formed new hydrogen bonds with the diphosphate of GDP in the presence of Mn^2+^. This conformational change suggested that Mn^2+^ coordination at the active site not only stabilizes the negative charge developing on the diphosphate group but also facilitates substrate binding.

### Nucleotide binding

**Figure 3:**
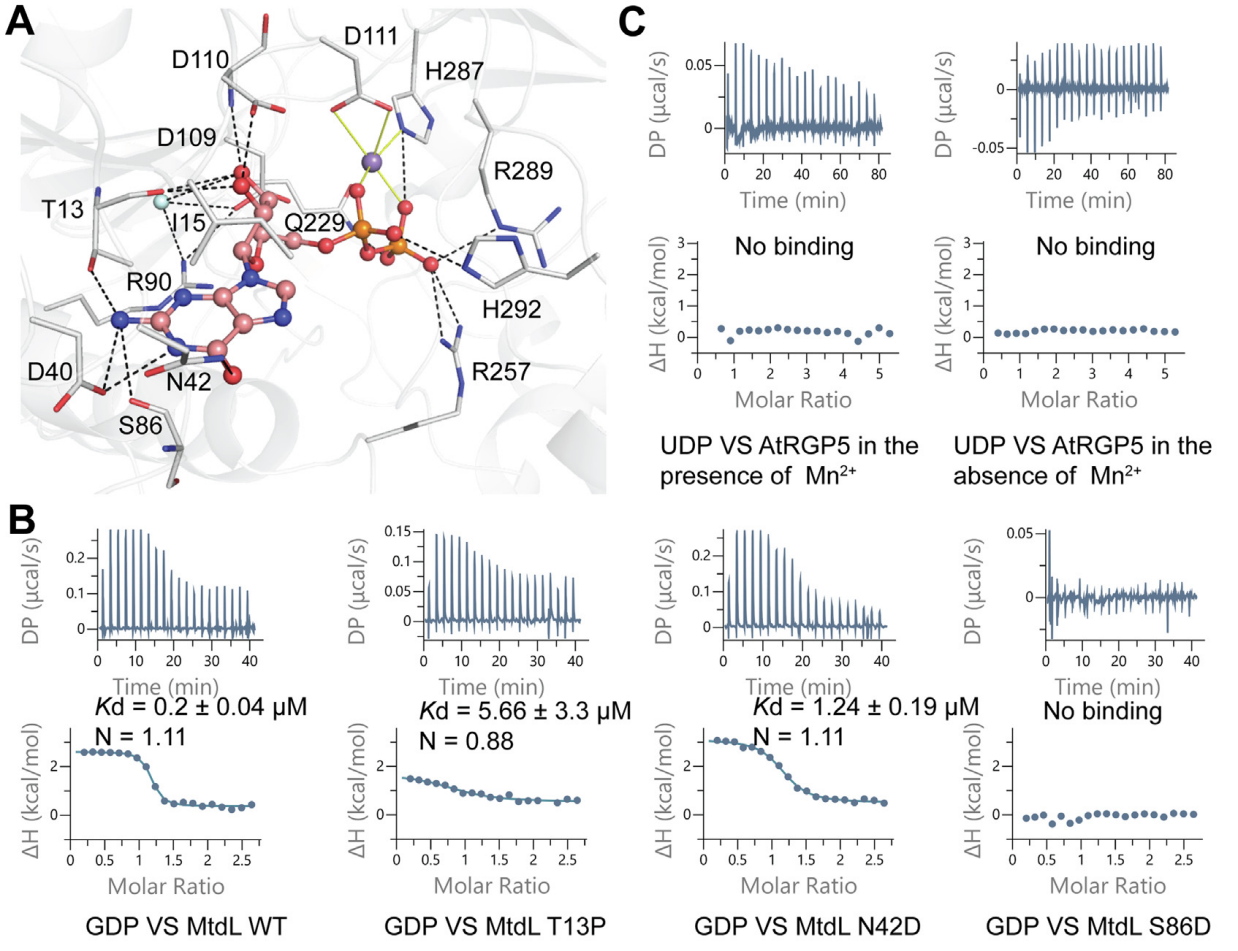
**Binding site for GDP of MtdL and key residues for base selectivity.***A*, hydrogen bonds to GDP and Mn^2+^-coordination bonds are represented by *black dashed* and *yellow full lines*, respectively, in the crystal structure of GDP·Mn^2+^·MtdL. *B*, isothermal titration calorimetry titration and fitting curves of WT MtdL and its T13P, N42D, and S86D variants with GDP. *C*, isothermal titration calorimetry titration and fitting curves of recombinant AtRGP5 with UDP in the presence and absence of Mn^2+^ ions.

Nucleotide binding details are shown in Figure 3*A*. In the MtdL·Mn^2+^·GDP structure, the α- and β-phosphates of GDP interact with the side chains of Asp111 and His287 *via* a Mn^2+^ ion. In addition, β-phosphate forms hydrogen bonds with the side chains of Gln229, Arg257, His287 and Arg289, while α-phosphate forms hydrogen bonds with the side chain of His292. For the ribose binding, the main chain oxygen of Thr13 hydrogen bonds to O2∗ and O3∗ of ribose. Besides, O2∗ hydrogen bonds to the carboxylate of Asp110, while O3∗ hydrogen bonds to the side chains of Arg90 and Asp109 *via* a water molecule, as well as the main chain nitrogen of Asp110. For the base binding, the guanine stacked against the side chain of Ile15. Meanwhile, O6 and N1 of guanine hydrogen bonds to the side chains of Asn42 and Asp40, respectively. N10 of guanine forms hydrogen bonds with the side chains of Asp40 and Thr13, as well as the main chain oxygen of Ser86.

Sequence alignment of some GT75 family members (Fig. S3) indicated that Ile15 and Asp40 are conserved among MtdL and RGPs, whereas Thr13, Asn42, and Ser86 are nonconserved. Bacterial MtdL and Hyg20 select GDP-sugars as substrates (2), whereas plant RGPs usually select UDP-sugars as substrates (3). Thus, we speculated that the nonconserved Thr13, Asn42, and Ser86 might be critical for base specificity. To validate this assumption, ITC experiments were performed using GDP titrated to WT MtdL and its T13P, N42D, and S86D variants (Fig. 3*B*). Here Thr12, Asn42, and Ser86 of MtdL were mutated to their corresponding residues in active RGP members. The WT protein showed the highest binding affinity to GDP with a *K*_d_ of approximately 0.2 μM. The N42D mutant displayed an approximately moderately reduced binding affinity (*K*_d_ ≈ 1.24 μM), suggesting a stronger hydrogen bond of an asparagine than an aspartate at residue 42 with O6 on guanine. The T13P mutant displayed a significantly reduced binding affinity (*K*_d_ ≈ 5.66 μM), indicating the contribution of the hydrogen bond between Thr13 and guanine N10 to base recognition. In particular, the S86D mutation completely prevented GDP binding, implying that the side chain of an aspartate at residue 86 might flip back to the base due to electrostatic repulsion with Asp87 and cause steric hindrance to guanine binding. These results supported the speculation that residues corresponding to Thr13 and Ser86 of MtdL should be key sites affecting the nucleotide base selectivity of GT75 enzymes.

We noticed that Mn^2+^ coordinating His287 and β-phosphate binding Arg257 are not conserved in inactive AtRGP5 but are highly conserved in active AtRGP1-3 (Fig. S3). This implied that catalytic inactivity of AtRGP5 might be attributed to its weak binding to UDP-sugar substrate. The results of ITC experiments supported the speculation that recombinant AtRGP5 showed undetectable binding to UDP in both the absence and the presence of Mn^2+^ ions (Fig. 3*C*).

### Sugar binding

To obtain the GDP-sugar complex structure of MtdL, we first validated the GDP-L-Fucp mutase activity of MtdL using ultrahigh-performance liquid chromatography (UPLC) with a hydrophilic interaction chromatography (HILIC) amide column along with time-of-flight mass spectrometry (TOF-MS). The *t*_R_ of GDP-L-Fucp was found to be 5.4 min, and a new peak representing product at *t*_R_ ≈ 4.8 min became evident compared with the negative control replacing the MtdL enzyme with its dissolved buffer (Fig. S4*A*). The product mass ([M-H]^-^ *m/z* = 588.0778) was identical to that of GDP-L-Fucp (Fig. S4*C*), suggesting that MtdL should interconvert GDP-L-Fucp to GDP-L-Fucf referring to the previous report (2). We later purified the product through a preparative amide column (Fig. S4*D*), soaked the crystal of MtdL-S228A·Mn^2+^ with this product, and observed its electron density in the crystal structure (Fig. S5*F*), further confirming that this product is GDP-L-Fucf. In contrast, when using GDP-D-Glcp as substrate, no product peak was observed, whether on an amide column (Fig. S6*A*) or on a C18 column (Fig. S6*B*).

**Figure 4:**
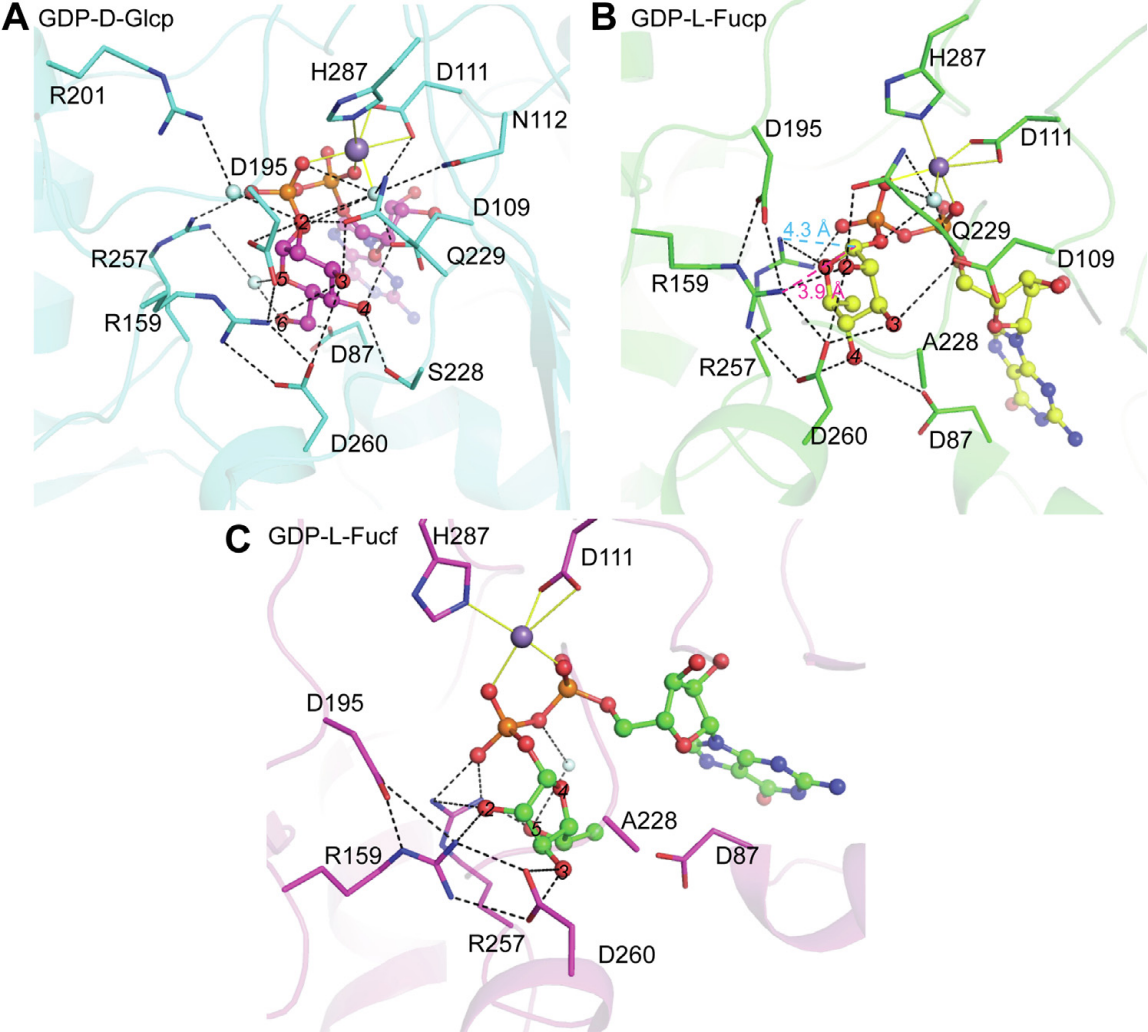
**Binding sites for different GDP-sugars of MtdL.***A*, residues hydrogen bonding to the glucose moiety are shown in the crystal structure of GDP-D-Glcp·Mn^2+^·MtdL. *B*, residues hydrogen bonding to the fucopyranose moiety are shown in the crystal structure of GDP-L-Fucp·Mn^2+^·MtdL. The distances of Arg159 and Arg257 to the anomeric carbon are highlighted. *C*, residues hydrogen bonding to the fucofuranose moiety are shown in the crystal structure of GDP-L-Fucf·Mn^2+^·MtdL. *A*–*C*, the oxygens on the sugar moieties are numbered. Water molecules are shown in cyan spheres.

The complex structure of MtdL bound to Mn^2+^ and a substrate analogue GDP-D-Glcp at 2.0 Å resolution was obtained by soaking the crystal of MtdL containing 0.5 mM MnCl_2_ with 5 mM GDP-D-Glcp. This structure contains two chains per ASU that can be superimposed with an RMSD of 0.106 A˚ over 327 Cα atoms. The D-Glcp moiety clearly defined by electron density is within the hydrogen bonding distance of several residues (Figs. 4*A* and S5, *A* and *D*). Its C2-OH hydrogen bonds to the side chains of Asp195 and Gln229 and forms water-mediated hydrogen bonds with Asp109, Asp111, and Asn112. Its C3-OH hydrogen bonds to the side chains of Arg159, Gln229, and Asp260. Its C4-OH hydrogen bonds to the side chains of Asp109 and Ser228. Its C6-OH hydrogen bonds to the side chain of Asp87 and forms a water-mediated hydrogen bond with the side chain of Arg257. Its C5O hydrogen bonds to the side chain of Arg257 through a water molecule.

To obtain the Mn^2+^·GDP-L-Fucp complex structure, we initially soaked the crystal of MtdL containing 0.5 mM MnCl_2_ with 5 mM GDP-L-Fucp. However, no electron density of the L-Fucp moiety was observed, possibly due to enzymatic catalysis toward GDP-L-Fucp. Then, the S228A mutant with decreased enzymatic activity was used for crystallization. The complex structure of MtdL-S228A·Mn^2+^·GDP-L-Fucp at 2.2-Å resolution was obtained by soaking the crystal of MtdL-S228A containing 0.5 mM MnCl_2_ with 5 mM GDP-L-Fucp and 0.5 mM MnCl_2_. This structure contains two chains per ASU that can be superimposed with an RMSD of 0.073 A˚ over 343 Cα atoms. The electron density for Mn^2+^ and the L-Fucp moiety could be seen in this structure (Fig. S5, *B* and *E*). Different interaction details of the L-Fucp moiety with MtdL were observed (Fig. 4*B*) in contrast with that of the D-Glcp moiety. Asp260 hydrogen bonds to the L-Fucp C2-OH, C3-OH, and C4-OH, simultaneously. Besides, the L-Fucp C2-OH hydrogen bonds to the side chains of Arg159 and Gln229. Its C3-OH hydrogen bonds to the side chains of Asp109. Its C4-OH hydrogen bonds to the side chains of Asp87. Its C5O hydrogen bonds to the side chain of Arg257.

Subsequently the Mn^2+^·GDP-L-Fucf complex structure at 2.4-Å resolution was obtained by soaking the crystals of MtdL-S228A containing 0.5 mM MnCl_2_ with 5 mM GDP-L-Fucf and 0.5 mM MnCl_2_. The GDP-L-Fucf compound was enzymatically synthesized from the MtdL-catalyzed reaction mixture using GDP-L-Fucp as a substrate and purified with a preparative amide column as mentioned before (Fig. S4*D*). This structure contains two chains per ASU that can be superimposed with an RMSD of 0.096 A˚ over 337 Cα atoms. The L-Fucf moiety and Mn^2+^ could be defined by the electron density in this structure (Fig. S5, *C* and *F*). The L-Fucf C2-OH hydrogen bonds to the side chains of Arg159 and Arg257. Its C3-OH hydrogen bonds to the side chain of Asp260. Its C5-OH hydrogen bonds to the side chain of Arg257 and forms a water-mediated hydrogen bond with the oxygen atom at the center of the diphosphate (Fig. 4*C*).

Superposition of the Mn^2+^·GDP and the three Mn^2+^·GDP-sugar complex structures indicated subtle structural changes between the four structures. Loop A in the three Mn^2+^·GDP-sugar complex structures exhibited only one conformation that is close to the diphosphate with clear electron densities for Arg289 and His292 (Fig. S5, *A*–*C*), in contrast to having two conformations in the Mn^2+^·GDP complex structure (Fig. S1*B*). Another two loops, loop B (residues 14–19) and loop C (residues 39–45) moved closer to GDP-sugar compared with GDP upon the ligand binding (Fig. S7*A*). Besides, a new Mn^2+^ coordination bond was observed, which is mediated by the carboxylate of Asp109 at the first position of the DXD motif *via* a water molecule in the GDP-D-Glcp and GDP-L-Fucp complex structures (Fig. 4, *A* and *B*). These structural changes indicated that sugar moiety binding further promoted the closure of the substrate binding pocket (Fig. S7, *C* and *D*), which should be ascribed to the enhanced hydrogen bonding network between the enzyme and nucleotide-sugar ligand. For example, Asp109, Gln229, and Arg257 hydrogen bond not only to GDP but also to the L-Fucp moiety. Asp109 and Gln229 hydrogen bond to the β-phosphate of GDP and coordinate to Mn^2+^ *via* a water (Fig. 4*B*). Arg257 directly hydrogen bonds to the β-phosphate (Figs. 3*A* and 4B). Meanwhile, Asp109, Gln229, and Arg257 hydrogen bond to the L-Fucp-C3-OH, -C2-OH, and -C5-O, respectively (Fig. 4*B*). Interactions with GDP were similar, and all the substrate-binding residues were superimposed well among the four GDP/GDP-sugar-bound structures (Fig. S7*B*). However, binding details with the three sugar moieties were different, which should dictate the different activities of MtdL toward the three types of GDP-sugars.

### Mutational analysis of the sugar-binding site

**Figure 5:**
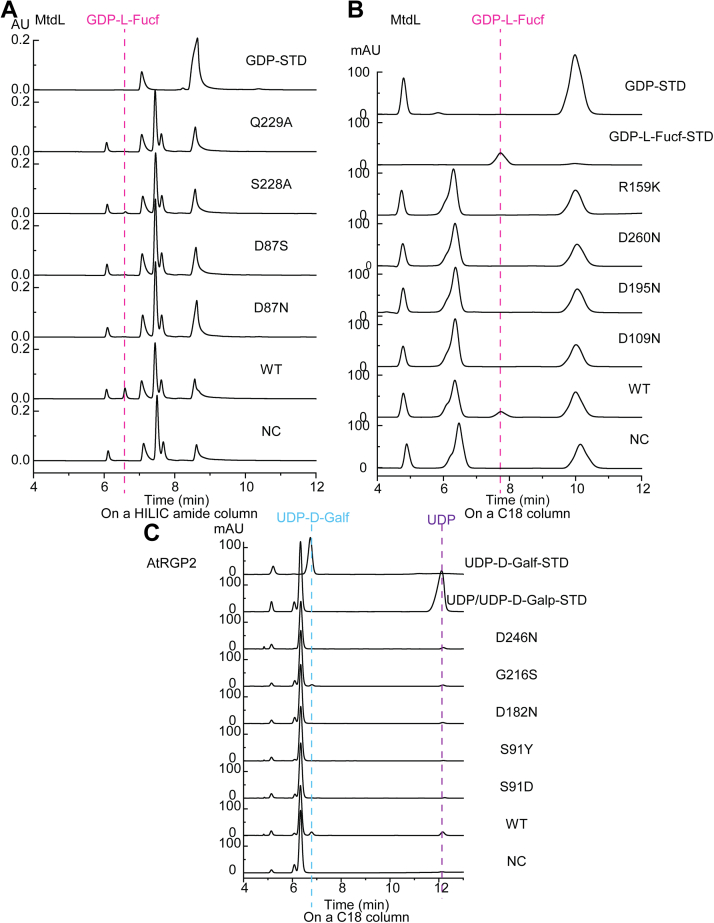
**Mutational analysis of the sugar-binding site.***A*, ultrahigh-performance liquid chromatography analyses on a hydrophilic interaction chromatography amide column of the *in vitro* reaction mixtures catalyzed by MtdL WT and its D87N, D87S, S228A, and Q229A mutants using GDP-L-Fucp as substrate. *B*, HPLC analyses on a C18 column of the *in vitro* reaction mixture catalyzed by MtdL WT and its D109N, R159K, D195N, and D260N mutants using GDP-L-Fucp as substrate. *C*, HPLC analyses on a C18 column of the *in vitro* reaction mixtures catalyzed by AtRGP2 WT and its S91D, S91Y, D182N, G216S, and D246N mutants using UDP-D-Galp as substrate.

To examine the roles of residues surrounding the sugar moiety, we purified D87N, D87S (mutated to the corresponding residue in active AtRGP1-3), D109N, D195N, D260N, S228A, Q229A, R159K, and R257K variants of MtdL and examined their activities toward GDP-L-Fucp in comparison with that of the WT. Enzymatic product analyses using UPLC with an amide column indicated that, except for the S228A mutant whose mutase activity diminished about 79% (on the basis of HPLC peak integrations), the D87N, D87S, and Q229A mutants showed no discernible mutase activity (Fig. 5*A*). We further assessed the mutase activity of other MtdL variants on a C18 column by comparison with the GDP-L-Fucf standard, which was at a *t*_R_ of approximately 7.7 min on a C18 column (Fig. 5*B*). The MtdL WT afforded a product peak at *t*_R_ ≈ 7.7 min, whereas no product could be detected for the D109N, D195N, D260N, R159K, and R257K mutants (Figs. 5*B* and S8*A*). Moreover, fluorescent thermal shift assay showed that MtdL melts with a sharp transition at *T*_m_ ≈ 61 °C. The mutations of D87N, D87S, D109N, Q229A, and R257K surrounding the sugar moiety (Figs. 4*B* and S7*D*) did not appreciably alter the observed *T*_m_ of the protein (Fig. S9, *A* and *B* and Table S2), which indicated that these mutations have little impact on the overall folding and the stability of MtdL. Although the mutants of R159K, D195N, and D260N located at the bottom of the substrate-binding pocket (Figs. 4*B* and S7*D*) showed relatively high initial fluorescence signal and anomalous melting at the initial stage (Fig. S9, *C* and *D*), indicative of temporary interaction between the SYPRO Orange dye and these variants at lower temperatures, these variants also melt with a transition at *T*_m_ ≈ 60 °C (Fig. S9*D* and Table S2), which suggested the thermal unfolding and overall structure might not be significantly perturbed by these three mutations. Because Asp109, Gln229, and Arg257 interact with both the GDP and the sugar moieties, the almost abrogated mutase activity of the D109N, Q229A, and R257K mutants might be due to significantly decreased binding of these mutants to GDP-sugar substrate. Similarly, reduced mutase activity of the S228A mutant should be caused by failure of stabilizing the L-Fucp moiety. Mutation of Arg159, corresponding to the conserved autoglycosylated arginine residue in plant UAMs/RGPs, to a lysine abrogated the mutase activity of MtdL, which is consistent with the vital role of this arginine in mutase activities of OsUAM1 (7), OsUAM3 (7) and HvUAM1 (8). In addition, the D87N, D87S, D195N, and D260N mutations all abolished the mutase activity of MtdL indicating critical roles of the three conserved aspartates surrounding the sugar moiety for catalysis.

Later, we examined the roles of the corresponding residues in AtRGP2 through HPLC analyses with a C18 column (Fig. 5*C*). Previous reports suggested that OsUAM1 can catalyze not only interconversion between UDP-D-Galp and UDP-D-Galf (12) but also autoglycosylation using UDP-D-Galp as substrate (6). Since AtRGP2 displayed 85.16% sequence identity with OsUAM1, we tested whether UDP-D-Galp could also induce both mutase and autoglycosyltransferase activities in recombinant AtRGP2. First, we assessed the AtRGP2 product mass against UDP-D-Galp using UPLC with a HILIC amide column along with TOF-MS. The *t*_R_ of UDP-D-Galp was found to be 8.6 to 8.7 min, and a new peak representing product at *t*_R_ ≈ 7.6 min became evident compared with the negative control replacing the AtRGP2 enzyme with its dissolved buffer (Fig. S10*A*). Similar to what MtdL showed, this product mass ([M - H]^-^ *m/z* = 565.0482) was the same as that of UDP-D-Galp ([M - H]^-^ *m/z* = 565.30) (Fig. S10, *B* and *C*), implicating that AtRGP2 should have mutase activity against UDP-D-Galp referring to the previous report (12). We also assessed the AtRGP2 product against UDP-D-Galp by using a C18 column. The *t*_R_ of the UDP standard was found to be 12.1 min, and a new product at *t*_R_ ≈ 12.1 min became evident in contrast with the negative control (Fig. 5*C*), suggesting AtRGP2 having glycosyltransferase activity against UDP-D-Galp. Meanwhile, the *t*_R_ of UDP-D-Galf standard, which was enzymatically synthesized from the AtRGP2-catalyzed reaction mixture using UDP-D-Galp as substrate and purified with a preparative amide column (Fig. S10*D*), was found to be approximately 6.8 min. This retention time corresponds to another product peak of AtRGP2-catalyzed reaction using UDP-D-Galp as substrate on a C18 column (Fig. 5*C*), implicating the dual activities of AtRGP2 against UDP-D-Galp. In contrast, only the product peak corresponding to UDP was observed from AtRGP2-catalyzed reaction using UDP-D-Glcp as substrate on a C18 column (Fig. S11), indicating that AtRGP2 shows only glycosyltransferase activity against UDP-D-Glcp. We then constructed S91D, S91Y (mutated to the corresponding residue in inactive AtRGP5), D182N, G216S, and D246N mutants of AtRGP2 with its Ser91, Asp182, Gly216, and Asp246 corresponding to Asp87, Asp195, Ser228, and Asp260 of MtdL, respectively. Except for the G216S mutant whose UDP-D-Galf product diminished about 54% (on the basis of HPLC peak integrations), the S91D, S91Y, D182N, and D246N mutants showed no discernible UDP-D-Galf product (Fig. 5*C*). At the same time, the side product of UDP seemed to be reduced to different degrees by these mutations.

### Oligomerization

MtdL is a tetramer as a dimer of dimers as mentioned before (Figs. 1, *A* and *B* and S2). Four dimerization interfaces mediate tetramer formation. Two are composed of Leu295, Leu298, and Val302 forming hydrophobic interactions. The other two are composed of Glu341 and Arg358 forming hydrogen bonding interactions. Interestingly, the L295D/L298D/V302D mutant became a monomer, whereas the E341K mutant became a dimer through SLC analyses (Fig. 1*B*). These results biochemically verified the two kinds of dimerization interfaces and indicated the key role of the hydrophobic dimerization interface in tetramer formation of MtdL. Oligomerization of MtdL contributes not only to its overall structural stability but also to its enzymatic activity. The L295D/L298D/V302D mutant displayed obviously decreased mutase activity toward GDP-L-Fucp compared with the E341K mutant and the WT (Fig. 1*C*). This was possibly because this dimerization interface, which is on α11 following loop A, moderately restricted the dynamic movement of loop A to facilitate its substrate binding.

More importantly, the hydrophobic residues at the key dimerization interface of MtdL are conserved among plant GT75 members (Fig. S3), implicating that plant members might also depend on these residues to form oligomers. Consistent with this assumption, aspartate substitutions of F280, L283, and Y287 of AtRGP2 corresponding to L295, L298, and V302 of MtdL, respectively, change the protein oligomeric form from a higher to a lower level through size exclusion chromatography analyses (Fig. S12*A*). Meanwhile, the F280D/L283D/Y287D mutant of AtRGP2 displayed no discernible mutase activity compared with that of the WT (Fig. S12*B*), further indicating the critical role of this conserved hydrophobic dimerization interface in oligomerization of GT75 family members and the stimulating effect of oligomerization on mutase activities in this family.

## Discussion

### A possible catalytic mechanism of the GT75 family

**Figure 6:**
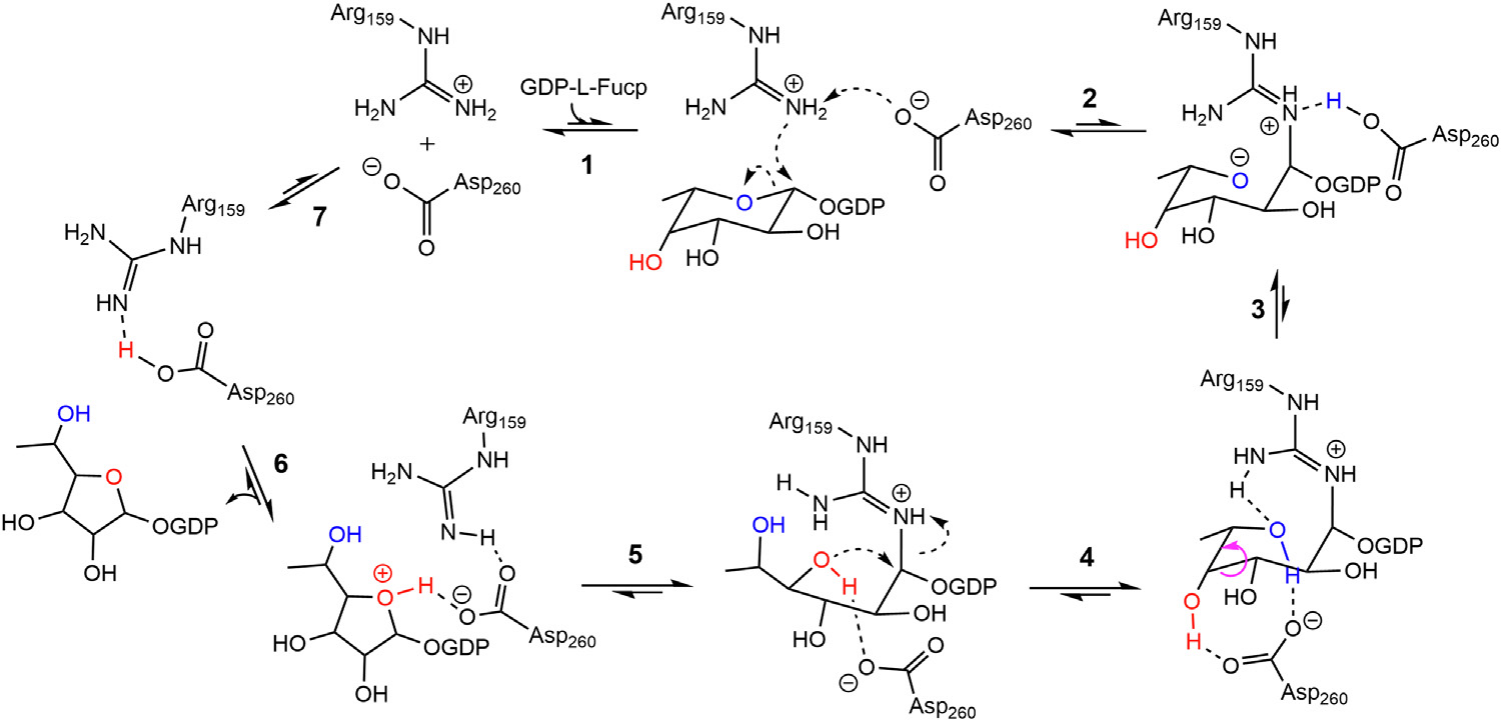
**Schematic diagram of a possible catalytic cycle of MtdL.** The mechanism of MtdL-catalyzed reaction cannot be unambiguously established by the currently available data. Here we proposed a possible catalytic cycle of MtdL. Asp260 is the best candidate to serve as the general base that deprotonates the acceptor Arg159 η-nitrogen for the nucleophilic attack of this nitrogen on L-Fucp-C1. Then the pyranose ring is opened and the proton is transferred from the carboxyl group of Asp260 to L-Fuc-C5O. Subsequently L-Fuc-C4O would attack at the L-Fuc-C1 for the furanose ring formation. This step might be performed by the rotation of the sugar C4-O and C5-O bonds accompanied by the transfer of the proton from C4-OH to the carboxyl group of Asp260. Finally the product GDP-L-Fucf is released from the active site and the proton is transferred back to the Arg159 η-nitrogen to complete the catalytic cycle.

The GT75 family is a unique bifunctional family. First, it represents one of three types of arginine glycosyltransferases currently discovered. Second, it is the sole glycosyltransferase family that can also achieve NDP-pyranose mutase activity. Currently available data do not allow the mechanism to be unambiguously established. Based on structural and mutational analyses and by analogy with the FAD-dependent UGM reaction mechanism (11, 18, 19, 20), we proposed a possible catalytic cycle of MtdL as shown in Figure 6.

Arg159 is located closer than Arg257 to the anomeric carbon with a distance of 3.9 and 4.3 Å, respectively, in the GDP-L-Fucp complex structure (Fig. 4*B*). Moreover, Arg257 interacts with both the sugar and the β-phosphate moieties of the substrate. It is a critical substrate-binding residue since no electron density was observed whether for the GDP or for the D-Glcp moiety in the structure of MtdL-R257K·Mn^2+^ cocrystallized with GDP-D-Glcp (Fig. S8, *B* and *C*), consistent with abrogated mutase activity of R257K (Fig. S8*A*). Therefore, the guanidinium group of Arg159 might be the best candidate to substitute for the role of FADH N5 as a nucleophile attacking the anomeric carbon.

Asp260 and Asp195 directly interact with the acceptor Arg159 η-nitrogen atoms (Fig. 4*B*), and asparagine substitutions of these two aspartates resulted in a significant loss of MtdL’s mutase activity toward GDP-L-Fucp (Fig. 5*B*). Nevertheless, Asp260, but not Asp195, is located in a position equivalent to that of the general base in the other two arginine glycosyltransferases EarP (21, 22) (Fig. S13) and NleB (23, 24). In addition, the product of UDP seemed to decrease more significantly from the reaction mixture catalyzed by AtRGP2-D246N compared with AtRGP2-D182N (Fig. S11) against UDP-D-Glcp, which induced only glycosyltransferase activity in AtRGP2. Therefore, Asp260 is the best candidate to serve as the general base that deprotonates the acceptor Arg159 η-nitrogen of MtdL itself for the nucleophilic attack of this nitrogen on L-Fucp-C1. One shared feature observed in inverting GTs of the GT-A fold is the position of the side chain of the catalytic base on the nucleoside-distal side as opposed to the nucleoside-proximal side of the sugar moiety observed in retaining GTs of the GT-A fold (25). The side chain of the predicted catalytic base Asp260 of MtdL is situated on the nucleoside-distal side of the sugar moiety (Fig. S14), further reflective of the inverting mechanism of MtdL. Asp195 might help modulate the orientation of the Arg159-L-Fuc adduct after direct attack of the Arg159 η-nitrogen to the L-Fuc-C1, facilitating transfer of the proton from the carboxyl group of Asp260 to the L-Fuc-C5O and opening of the pyranose ring.

Subsequently, the L-Fuc-C4-O^-^ would nucleophilic attack at the L-Fuc-C1 for the furanose ring formation. Asp260 might accept the proton from L-Fuc-C4-OH accompanied by direct attack of L-Fuc-C4-O^-^ to L-Fuc-C1 to generate the furanose form. The nearby Asp87 might assist Asp260 in deprotonating L-Fuc-C4-OH. In addition, the conserved Arg257 forms new hydrogen bonds with L-Fucf C2-OH and C5-OH (Fig. 4*C*), possibly facilitating ring contraction. We noted that L-Fucp C4-OH is positioned at a hydrogen bond distance (3.1 Å) with the carboxyl group of Asp260 in the MtdL·Mn^2+^·GDP-L-Fucp complex structure (Fig. 4*B*), whereas the D-Glcp C4-OH is positioned with a distance of 3.8 Å to the carboxyl group of Asp260 in the MtdL·Mn^2+^·GDP-D-Glcp complex structure (Figs. 4*A* and S13*B*). The shorter distance of L-Fucp C4-OH compared with that of D-Glcp C4-OH to the putative catalytic base might dictate the mutase activity of MtdL toward GDP-L-Fucp instead of GDP-D-Glcp. By further comparative structural analysis of the active sites of MtdL and the arginine glycosyltransferase EarP (Fig. S13*A*), we speculated that the pyranose C4-OH being located close enough to the base catalyst of a GT75 enzyme might decide which NDP-pyranose induces mutase activity of the enzyme. In other words, the conserved aspartate corresponding to Asp260 of MtdL might play multiple roles. One serves as the general base that deprotonates the acceptor η-nitrogen atom of the conserved arginine, corresponding to Arg159 of MtdL, for the nucleophilic attack of this nitrogen to the anomeric carbon to generate the arginine-sugar adduct. The second is to give the proton to the sugar C5-O for the pyranose ring opening. The third is to accept the proton from the sugar C4-OH accompanied by the attack of the C4-O^-^ to the anomeric carbon for the furanose ring generation. Finally, the newly obtained proton by Asp260 would be transferred back to Arg159 η-nitrogen and release of the product GDP-L-Fucf from the active site would complete the catalytic cycle.

### Our study has laid a foundation for the utilization of GT75 family members

GT75 family members have two potential applications: (i) GT75 family members can be applied in valuable NDP-furanose synthesis. The glycosylation of natural products often dramatically influences the biological and/or pharmacological activities of the parental metabolites. Changing structures of the sugar moieties of many glycosylated natural products can have a profound impact on the biological properties of the parental compounds. Many furanoses, such as L-Galf, L-Araf, and L-Fucf, have been found to be crucial building blocks in some important naturally bioactive compounds (26). In addition, structural analogs of furanosyl glycoconjugates would be of great interest for the elucidation of the role of furanosides in innate recognition events upon the host–pathogen interaction. However, the limited availability of NDP-furanoses greatly hampers the development of novel furanosyl glycoforms of secondary metabolites with useful biological activity. The chemical synthesis and purification of NDP-furanoses are notoriously inefficient (27). GT75 family members can be harnessed in enzymatic synthesis of various NDP-furanoses by combining a sugar-1-phosphate kinase, which catalyzes the formation of pyranosyl-1-phosphate from monosaccharide and ATP, and an NDP-sugar pyrophosphorylase (also called a nucleotidyltransferase), which catalyzes the formation of NDP-pyranose from pyranosyl-1-phosphate and NTP (28, 29). Compared with FAD-dependent UGMs, divalent cation-dependent GT75 members showed a broader substrate scope. For example, bacterial members, such as MtdL and Hyg20, can produce GDP-L-Fucf and GDP-L-Galf from GDP-L-Fucp and GDP-L-Galp, respectively. Plant members, such as OsUAM1 and AtRGP2, can produce UDP-L-Araf and UDP-D-Galf from UDP-L-Arap and UDP-D-Galp, respectively. Some degree of substrate promiscuity of the GT75 family would make them more suitable for biotechnological applications. GT75 members can also be engineered to broaden their substrate specificity and enhance their mutase activity and stability for biotechnological applications such as directed evolution of MtdL based on its crystal structures in complex with different GDP-sugars. (ii) GT75 family members offer a tool to control plant cell wall composition. Lignocellulosic biofuel production is inhibited by the plant cell wall’s natural recalcitrance against enzymatic degradation. It has been shown that downregulation of a UAM gene in switchgrass produced a decrease in arabinoxylan with a concurrent increase in lignin, while sugar release efficiency was not negatively affected (30). The structure of MtdL provides a template for further engineering plants *via* transgenic manipulation of designed UAM mutants. In summary, our study not only has paved the way for a better understanding of the mechanism behind the dual activities of the GT75 family but also has served as a starting point for engineering GT75 enzymes to facilitate the production of various NDP-furanoses and furanosides.

## Experimental procedures

### Cloning, expression, and purification

A codon-optimized MtdL (UniProtKB accession number G8HX37) gene was synthesized and cloned into a modified pET28a vector, in which thrombin protease site was substituted for tobacco etch virus (TEV) cleavage site. A codon-optimized AtRGP2 (UniProtKB accession number Q9LFW1) gene was synthesized and cloned into pET32a. A codon-optimized AtRGP5 (UniProtKB accession number Q9FFD2) gene was synthesized and cloned into a modified pGEX-4T1 vector, in which thrombin protease site was substituted for TEV cleavage site. All mutants were generated using the Takara MutanBEST kit. All clones were verified by DNA sequencing.

The recombinant plasmids were transformed into *Escherichia coli* BL21-Gold (DE3) cells. The transformed cells were grown in LB medium at 37 °C until the absorbance at 600 nm reached about 0.8. Then the proteins were induced with 0.1 mM isopropyl β-D-1-thiogalactopyranoside (IPTG) at 16 °C for 24 h. The cells were collected by centrifugation, resuspended, and sonicated in buffer A (20 mM Tris-HCl, pH 8.0, 500 mM NaCl). After centrifugation, the supernatants for MtdL, AtRGP2, and their mutants were loaded on a nickel-sepharose column, and the proteins were purified and eluted with buffer A containing an imidazole gradient. MtdL without His_6_-tag for crystallization was purified using a nickel-sepharose column, treated with TEV to remove His_6_ tag, and further purified on a nickel-sepharose column (collecting wash fraction with 20 mM imidazole). The TEV protease was also removed by this step since it contains a His_6_-tag. The supernatants for AtRGP5 and its mutants were loaded on a glutathione-sepharose column, and the proteins were purified with buffer A containing 1% Triton X-100, followed by passage through the column after TEV cleavage on the column to remove GST-tag. The TEV protease containing a His_6_-tag was further removed by a nickel-sepharose column. All the proteins were further purified on a Superdex 200 HiLoad 16/60 column preequilibrated with buffer B (20 mM Tris-HCl, pH 8.0, 200 mM NaCl). The purified proteins were then concentrated for subsequent analysis.

To prepare SeMet-derivative MtdL, *E. coli* was grown in 1 L of LR medium (a minimal medium described by LeMaster and Richards) at 37 °C until the absorbance at 600 nm reached about 0.8. Then, 60 mg SeMet and 50 mg each of threonine, lysine, phenylalanine, leucine, isoleucine, and valine were added as solids to the growing culture. After continued growth for 1 h at 16 °C, IPTG was added to 0.1 mM to the culture, and the procedure was completed as usual. Purification was performed essentially as for native protein.

### Crystallization

All the crystals were grown using the sitting drop vapor diffusion method with 1 μl of protein solution mixed with 1 μl of reservoir solution at 10 °C. Crystals of SeMet-derivative MtdL in complex with GDP were grown by cocrystallizing SeMet-derivative MtdL without His_6_-tag and GDP at a molar ratio of 1:3 with reservoir buffer A (1.0 M lithium chloride, 0.1 M citric acid, pH 5.0, 10% (w/v) PEG6000). Crystals of MtdL in complex with Mn^2+^ ions and GDP were obtained by cocrystallizing untagged MtdL containing 0.5 mM MnCl_2_ with GDP at a molar ratio of 1:3 with reservoir buffer B (0.2 M ammonium sulfate, 0.1 M tri-sodium citrate, pH 5.6, 15% (w/v) PEG 4000). Crystals of MtdL in complex with Mn^2+^ ions and GDP-D-Glcp were obtained by crystallizing untagged MtdL containing 0.5 mM MnCl_2_ with reservoir buffer C (0.1 M tri-sodium citrate pH 5.6, 10% (w/v) PEG 4000, 10% (w/v) isopropanol) and then soaking the MtdL·Mn^2+^ crystals in reservoir buffer C supplemented with 5 mM GDP-D-Glcp for 10 min. Crystals of MtdL-S228A in complex with Mn^2+^ ions and GDP-L-Fucp were grown by crystallizing N-terminally His_6_-tagged MtdL-S228A supplemented with 0.5 mM MnCl_2_ with reservoir buffer D (0.2 M ammonium sulfate, 0.1 M Tris, pH 7.5, 20% (w/v) PEG 5000 MME) and then soaking the MtdL-S228A·Mn^2+^ crystals in reservoir buffer D supplemented with 5 mM GDP-L-Fucp and 0.5 mM MnCl_2_ for 10 min. Crystals of MtdL-S228A in complex with Mn^2+^ ions and GDP-L-Fucf were grown by soaking the MtdL-S228A·Mn^2+^ crystals in reservoir buffer D supplemented with 5 mM GDP-L-Fucf and 0.5 mM MnCl_2_ for 10 min. GDP-L-Fucf was enzymatically synthesized from the MtdL-catalyzed reaction mixture using GDP-L-Fucp as substrate and purified with a preparative amide column as mentioned below.

### Data collection, structure determination, and refinement

X-ray diffraction data were collected at 100 K at the BL19U1 beamline at the Shanghai Synchrotron Radiation Facility, China. All the crystals were cryoprotected with a reservoir solution containing 25% glycerol. All the diffraction images were processed using XDS (31) and further scaled by Aimless in CCP4i software suite (32). The phase of the Se-Met derivative crystal was determined by the SAD method. The selenium sites were located by Shelx C/D software (33), the phase was calculated and improved by PHENIX.Autosol, and an initial model was automatically built by PHENIX.AutoBuild (34). The model of Se-Met derivative MtdL in complex with GDP was further refined by PHENIX.refine and Coot (35). The other structures were determined by molecular replacement in PHASER (36) with the structure of Se-Met derivative MtdL as the search model and were further refined by PHENIX.refine and Coot. TLS refinement was used in the late stage of the refinement. All data collection and refinement statistics are summarized in Table S1.

### Isothermal titration calorimetry

ITC assays were carried out on a MicroCal PEAQ-ITC calorimeter at 20 °C. The titration protocol for GDP titrated to MtdL consisted of a single initial injection of 1 μl, followed by 19 injections of 2 μl ligand (GDP) into the sample cell containing MtdL WT or its mutants in buffer B supplemented with 0.5 mM MnCl_2_, using an interval time of 2 min between injections. The titration protocol for UDP titrated to AtRGP5 consisted of a single initial injection of 1 μl, followed by 19 injections of 2 μl ligand (UDP) into the sample cell containing AtRGP5 in buffer B or in buffer B supplemented with 0.5 mM MnCl_2_, using an interval time of 4 min between injections.

### *In vitro* enzymatic assays

An assay for the activity of MtdL, AtRGP2, or their mutants was performed in a 200-μl reaction system consisting of 0.5 mM substrate and 0.25 mM MnCl_2_ in buffer C (50 mM NaH_2_PO_4_/Na_2_HPO_4_, pH 6.5), when 5 μM enzyme was added to initiate the reaction. Each reaction was carried out at 37 °C for 30 min. All reactions were quenched by heating to 100 °C for 2 min, and the reaction mixtures were then centrifuged at 14,000 rpm for 20 min to remove any precipitates. The remaining supernatants were then analyzed by analytical HPLC or UPLC. All HPLC analyses were carried out using Thermo Scientific UltiMate 3000 system with a C18 column (ZORBAX Eclipse XDB-C18 Analytical 4.6 × 250 mm, 5 μm). The isocratic mobile phase for all analytical HPLC separations consisted of 10% acetonitrile and 50 mM Na_2_HPO_4_/NaH_2_PO_4_ in ddH_2_O, pH 6.5, 10 mM tetrabutylammonium bromide at a flow rate of 0.8 ml/min and column temperature of 45 °C. Analytical UPLC was performed with Waters ACQUITY UPLC H-Class system with a HILIC column (ACQUITY UPLC BEH Amide 2.1 × 100 mm, 1.7 μm). The analytical UPLC solvent system consisted of solvent A (100 mM ammonium acetate in ddH_2_O, pH 6.6) and solvent B (100% acetonitrile). Samples were eluted at 0.2 ml/min and room temperature with 28% solvent A for 2 min, a linear gradient of 28% to 38% solvent A over 0.1 min, 38% solvent A for 15.9 min, followed by a linear gradient of 38% to 70% solvent A over 0.1 min, 70% solvent A for 2.9 min, a linear gradient of 70% to 28% solvent A over 0.1 min, and elution with 28% solvent A for 3.9 min. UV detection was carried out at 254 nm.

### Liquid chromatography–mass spectrometry

MtdL and AtRGP2-mediated reactions were analyzed by ultrahigh-performance liquid chromatography-electrospray ionization-tandem mass spectrometry (UPLC/ESI-Q-TOF-MS) to confirm the correct molecular weights for each substrate and product species. This process was conducted on a 1290 Infinity II LC coupled with a 6545XT AdvanceBio LC/Q-TOF system equipped with a Waters HILIC column (ACQUITY UPLC BEH Amide 2.1 × 100 mm, 1.7 μm). Each procedure, including gradients and detection for chromatographic separation, was carried out as analytical UPLC as noted above.

### Enzymatic synthesis and isolation of GDP-L-fucofuranose and UDP-D-galactofuranose

The enzyme reactions were carried out in a volume of 500 μl consisting of 3 mM GDP-L-Fucp or UDP-D-Galp, 0.25 mM MnCl_2_ in buffer C, and 40 μM enzyme (MtdL or AtRGP2). After incubation at 37 °C for 4 h, each reaction was quenched by heating to 100 °C for 2 min, and the sample was then centrifuged twice at 14,000 rpm for 20 min to remove any precipitates. Supernatants were then subjected to semipreparative HPLC with Waters Prep HPLC 2535 Quaternary Gradient Module using a Waters XBridge BEH Amide OBD Prep Column, 10 × 250 mm, 5 μm. By scaling up from analytical UPLC separation conditions, the gradient used for semipreparative HPLC was as follows: solvent A was maintained at 28% for 5 min, an increase to 38% solvent A over 0.25 min, maintained at 38% solvent A for 39.75 min, followed by an increase to 70% solvent A over 0.25 min, maintained at 70% solvent A for 7.25 min, a decrease to 28% solvent A over 0.25 min, and then equilibration at 28% solvent A for 9.75 min. Samples were eluted at a flow rate of 5 ml/min and at room temperature. UV detection was carried out at 254 nm. The purified products were lyophilized at −40 °C for 20 h, and the resultant white powders were stored at −80 °C.

### Static light scattering

SLC was performed using an Akta Pure L system (GE healthcare) with a Superdex 200 Increase column (GE healthcare). The system was coupled on-line to an eight-angle multiangle static light scattering detector (DAWN HELEOS II, Wyatt Technology) and a differential refractive index detector (Optilab T-rEX, Wyatt Technology). Molar masses were determined using ASTRA 7.0.1 software.

### Thermal shift assay

Thermal shift assay (TSA) was carried out on LightCycler 480 Multiwell Plates 384 (Roche). A volume of 10 μl of TSA mix containing 0.2 mg/ml protein (in 20 mM Tris, pH 8.0, 200 mM NaCl, 0.5 mM MnCl_2_) and the fluorescent dye SYPRO orange (1:1000) was dispensed into each well. After 15 s of incubation at 25 °C, the increase of fluorescence at 580 nm (excitation 465 nm) during the 25 to 95 degree melting (0.01 °C/s) was measured. Each protein sample was monitored in triplicate. The *T*_m_ was determined from the first derivative of the melting curve.

## Data availability

The atomic coordinates for GDP·SeMet-MtdL, GDP·Mn^2+^·MtdL, GDP-D-Glcp·Mn^2+^·MtdL, GDP-L-Fucp·Mn^2+^·MtdL-S228A, GDP-L-Fucf·Mn^2+^·MtdL-S228A, and Mn^2+^·MtdL-R257K have been deposited in the RCSB Protein Data Bank with accession numbers 7XPR, 7XPS, 7XPT, 7XPU, 7XPV, and 8HL8, respectively.

## Supporting information

This article contains supporting information.

## Conflicts of interest

The authors declare that they have no conflicts of interest with the contents of this article.
