# Supplementary material for: Structures of the NDP-pyranose mutase belonging to glycosyltransferase family 75 reveal residues important for Mn2+ coordination and substrate binding
Source: J Biol Chem. 2023 Jan 13;299(2):102903. doi: 10.1016/j.jbc.2023.102903 (PMC9937993; doi:10.1016/j.jbc.2023.102903)
Supplement: Supplemental data [file mmc1.doc]

**Supporting Information for**

**Structures of the NDP-pyranose mutase belonging to glycosyltransferase family 75 reveal residues important for Mn^2+^ coordination and substrate binding**

**Xueqing Du^1#^, Xuan Chu^1#^, Ning Liu^1#^, Xiaoyu** **Jia^1^, Hui** **Peng^1^, Yazhong** **Xiao^1^, Lin** **Liu^1^, Haizhu Yu^3^, Fudong** **Li^2^, Chao He^1*^**

^1^Anhui Key Laboratory of Modern Biomanufacturing and School of Life Sciences, Anhui University, Hefei, Anhui 230601, China

^2^MOE Key Laboratory for Cellular Dynamics, School of Life Sciences, Division of Life Sciences and Medicine, University of Science and Technology of China, Hefei, China.

^3^Department of Chemistry and Centre for Atomic Engineering of Advanced Materials, Anhui Province Key Laboratory of Chemistry for Inorganic/Organic Hybrid Functionalized Materials, Anhui University, Hefei 230601, China.

^#^Both authors contributed equally to this work.

^*^Corresponding to Chao He, **E-mail**: chaohe@ahu.edu.cn


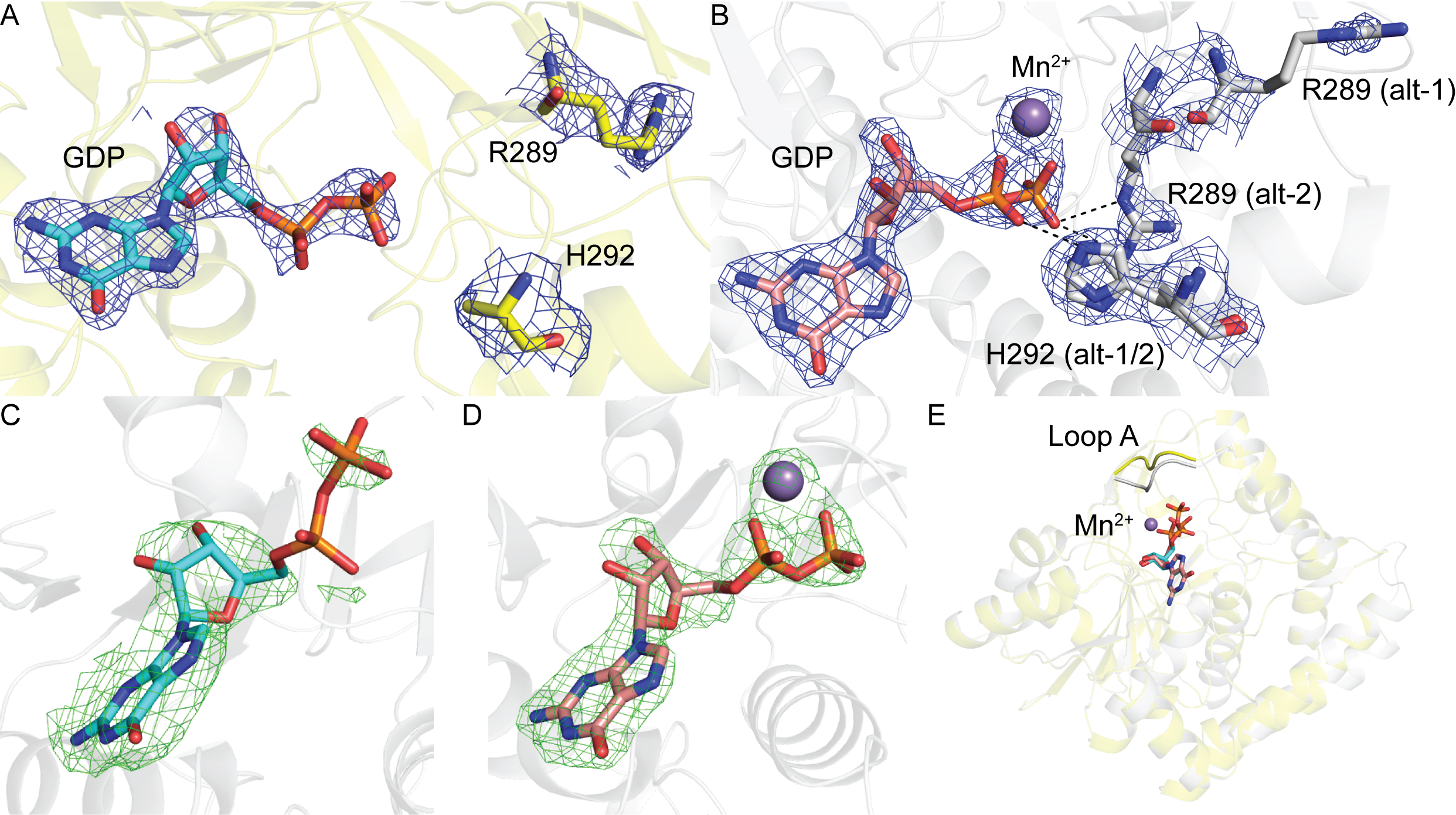


**Fig. S1.** (A) 2Fo-Fc electron density maps contoured at 1σ for GDP, residues Arg289 and His292 from loop A (residues 288-292, colored in yellow) and (B) Polder map contoured at 3σ for GDP in SeMet-MtdL·GDP structure without Mn^2+^ ions. (C) 2Fo-Fc electron density maps contoured at 1σ for GDP, Mn^2+^, residues Arg289 and His292 from loop A (colored in gray) and (D) Polder map contoured at 3σ for GDP and Mn^2+^ in MtdL·Mn^2+^·GDP structure. Arg289 and His292 displayed two conformations in this structure. (E) Superposition of GDP-bound structures of MtdL in the absence and in the presence of Mn^2+^ ions.


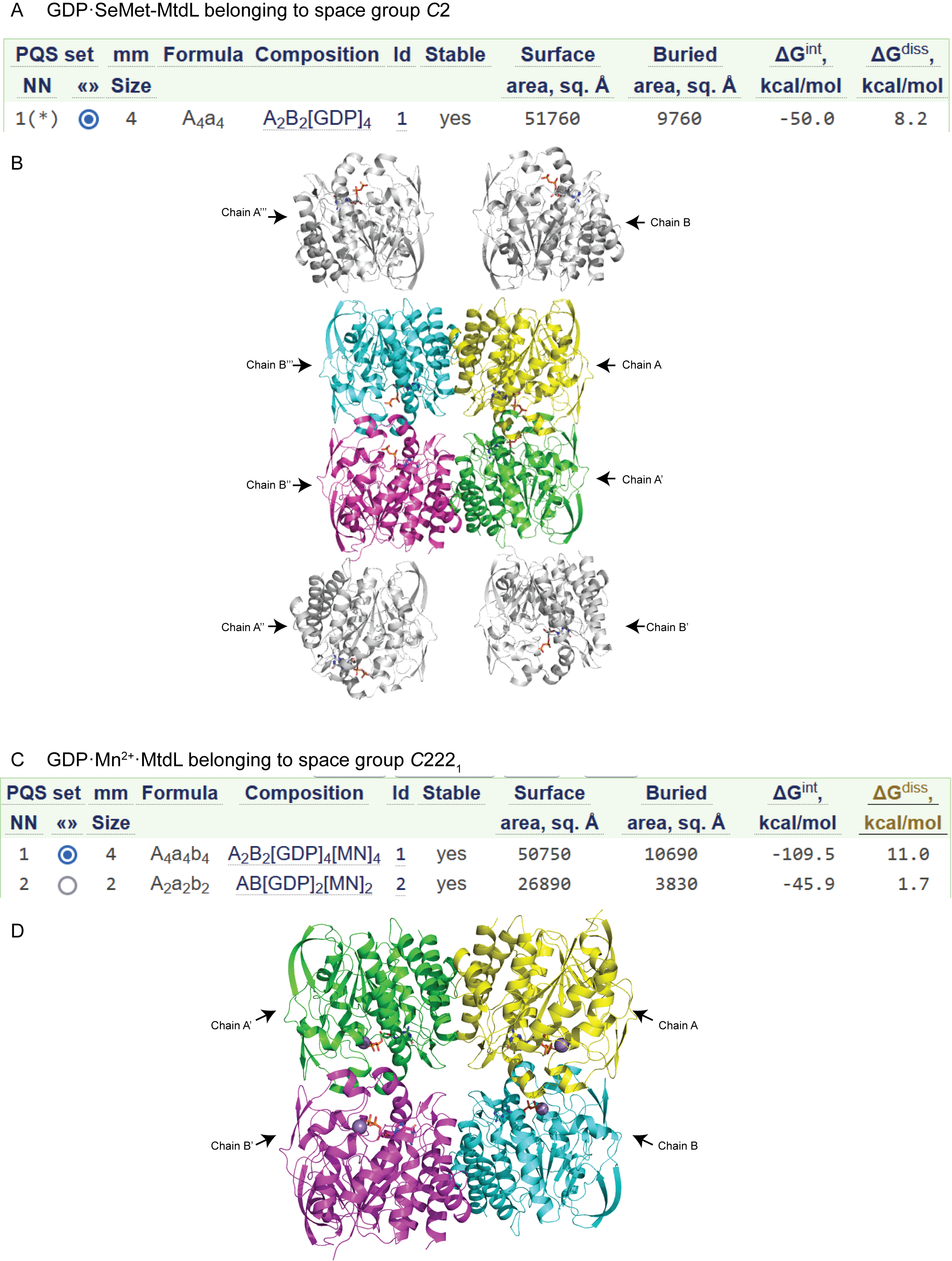


**Fig. S2.** Analysis of assemblies for MtdL crystal structures belonging to two different space groups. (A) Tetramer predicted by PISA for the crystal structure of GDP·SeMet-MtdL belonging to space group *C*2. (B) The whole tetramer structure was visualized by generating symmetric mates in PyMOL. There are two chains per asymmetric unit (ASU) (chain A and B, or chain A’ and B’, etc.). The four chains that constitute a tetramer come from four ASUs, respectively, in the crystal of GDP·SeMet-MtdL. (C) Tetramer predicted by PISA for the crystal structure of GDP·Mn^2+^·MtdL belonging to space group *C*222_1_. (D) The whole tetramer structure was visualized by generating symmetric mates in PyMOL. There are two chains per ASU (chain A and B, or chain A’ and B’). One ASU is half a tetramer in the crystal of GDP·Mn^2+^·MtdL.


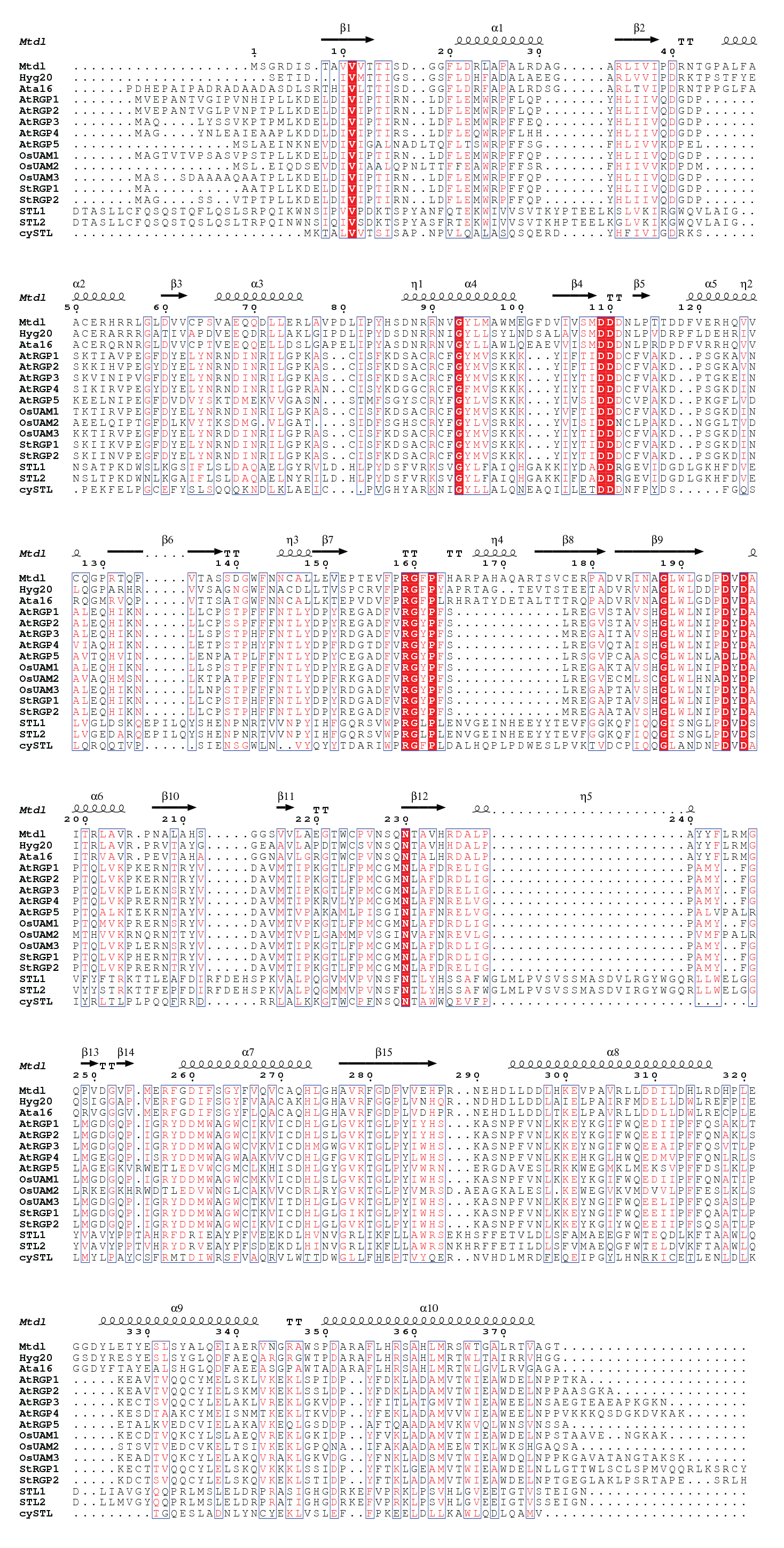


**Fig. S3.** Sequence alignment of some GT75 family members.


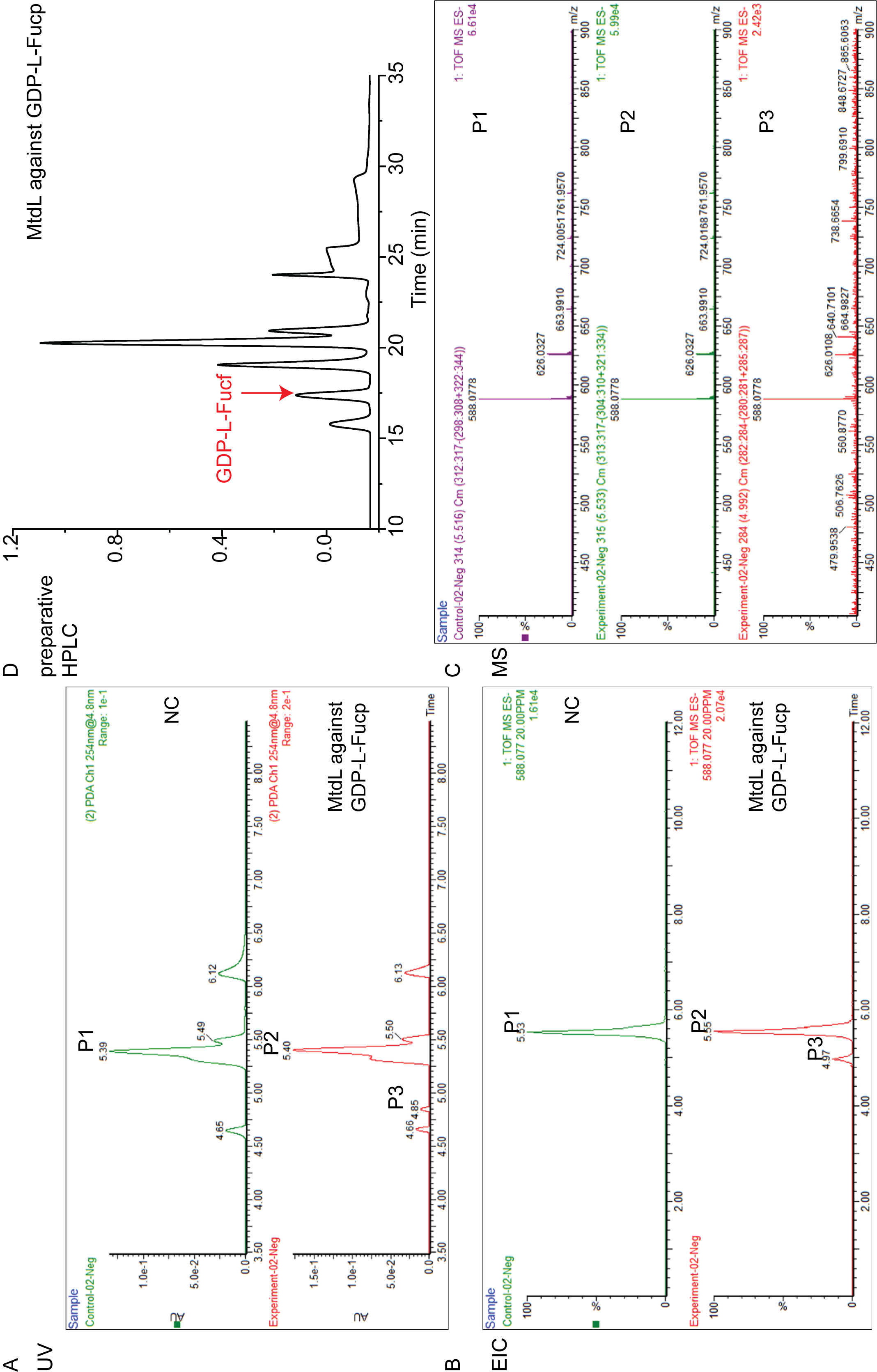


**Fig. S4.** UPLC analyses equipped with a HILIC amide column (A) and extracted ion chromatogram-mass spectrometry (EIC-MS) analyses (B and C) of MtdL-catalyzed reaction mixture in vitro using GDP-L-Fucp as substrate. (A) The above chromatogram labeled “NC” represents a negative control which replaced the MtdL enzyme with its dissolved buffer. The below chromatogram labeled “MtdL against GDP-L-Fucp” represents the reaction mixture catalyzed by MtdL using GDP-L-Fucp as substrate. Peak P1 represents the substrate GDP-L-Fucp in the negative control. Peaks P2 and P3 represent the remaining substrate and new enzymatic product in the MtdL-catalyzed reaction by comparison to the negative control above. (B) are extracted ion chromatograms of (A) at chosen mass-to-charge value. The delay time of ultraviolet (UV) detector and mass spectrometry detector here was about 0.1 min. (C) The above, middle and below mass spectra correspond to the samples in peaks P1, P2 and P3, respectively. The results indicated that the new enzymatic product corresponding to peak P3 ([M - H]^-^ m/z = 588.0778) had a mass almost identical to that of the substrate GDP-L-Fucp ([M - H]^-^ m/z = 588.34). (D) HPLC purification of the product GDP-L-Fucf of MtdL catalysis using a preparative amide column.


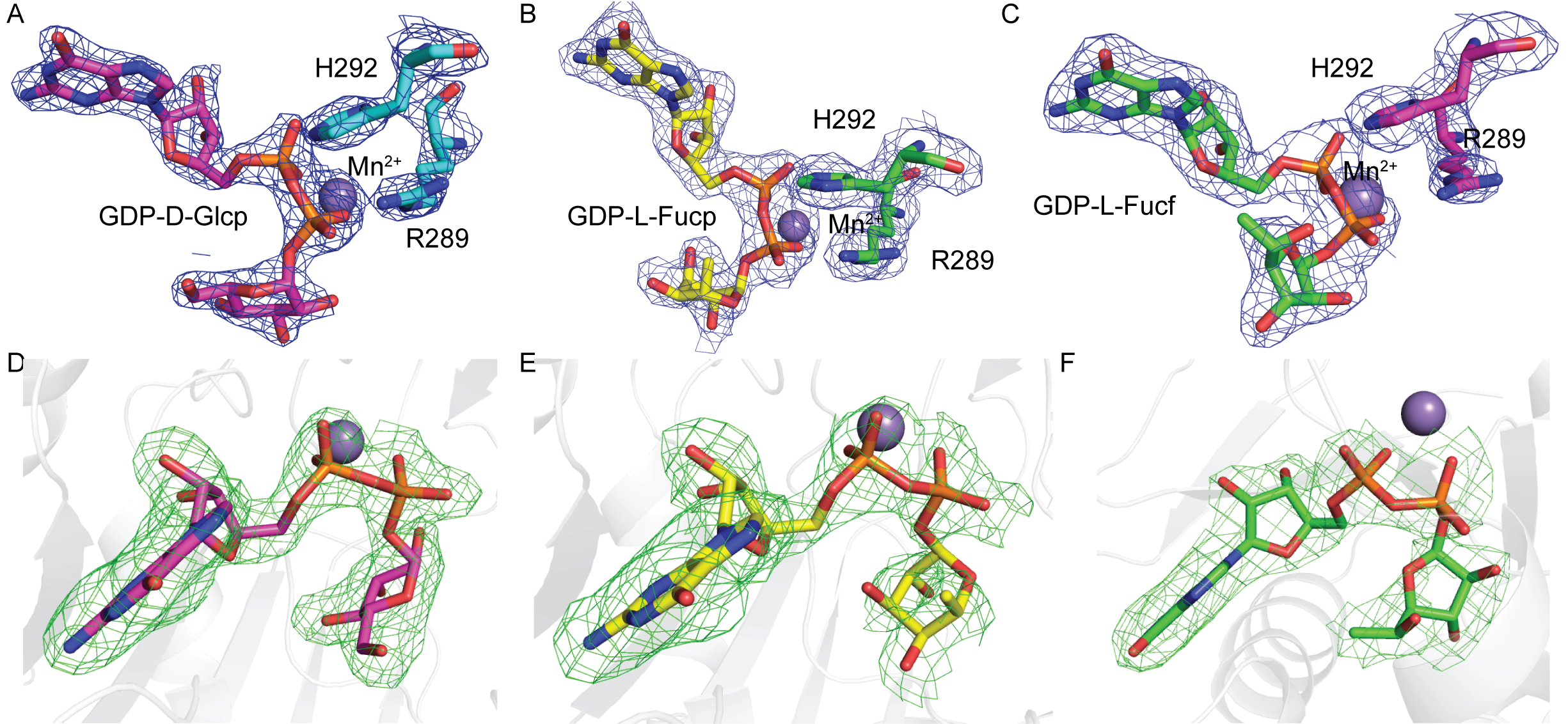


**Fig. S5.** (A-C) Fo-Fc electron density maps for GDP-D-Glcp, GDP-L-Fucp and GDP-L-Fucf, respectively, and residues Arg289 and His292 contoured at 1σ, as well as the Mn^2+^ ion contoured at 4σ, in the three GDP-sugar complex structures. (D-F) Polder maps contoured at 3σ for GDP-D-Glcp, GDP-L-Fucp and GDP-L-Fucf, respectively, in the three GDP-sugar complex structures.


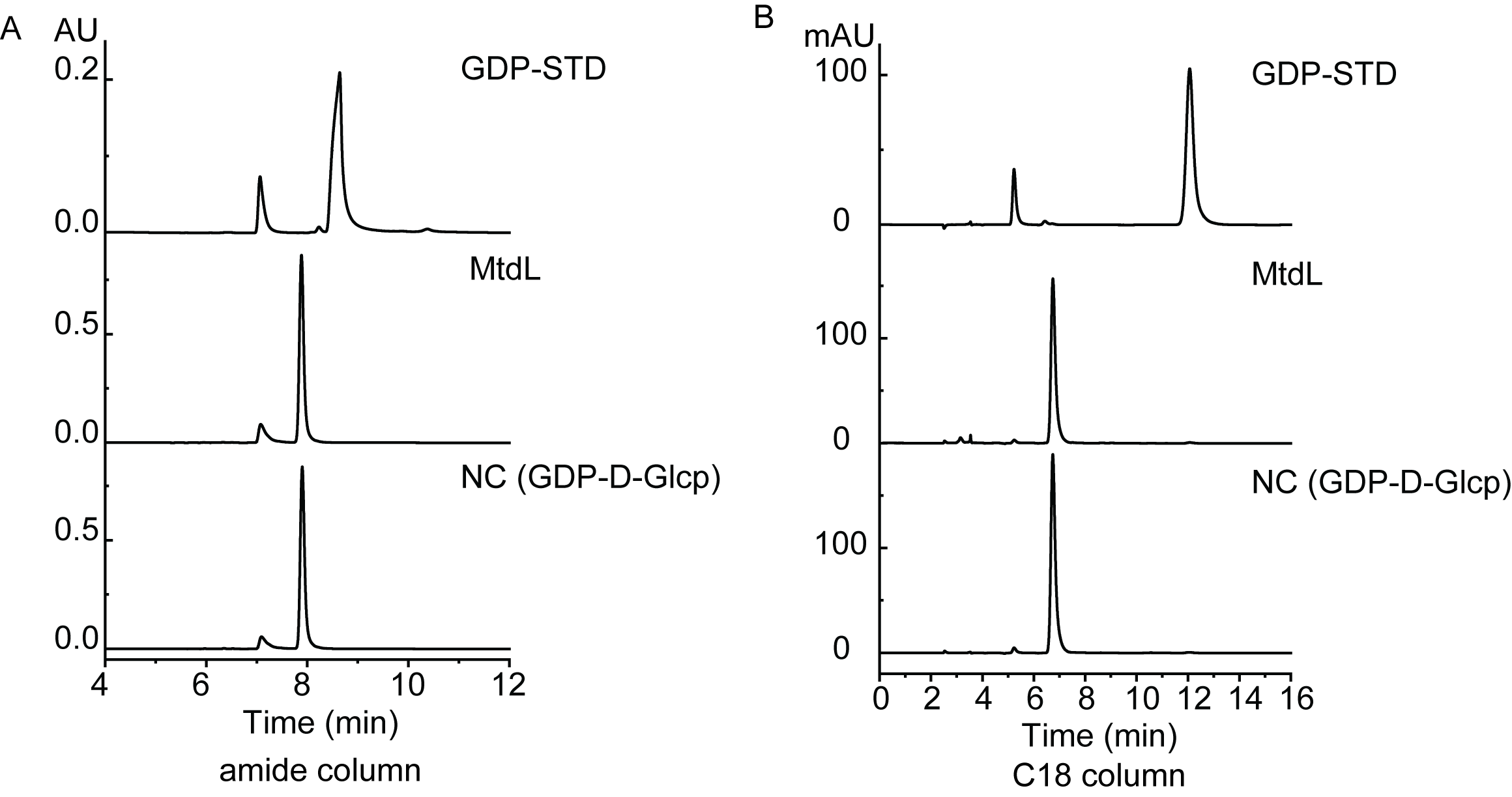


**Fig. S6.** UPLC analyses equipped with a HILIC amide column (A) and HPLC analyses equipped with a C18 column (B) of MtdL-catalyzed reaction mixtures using GDP-D-Glcp as substrate.


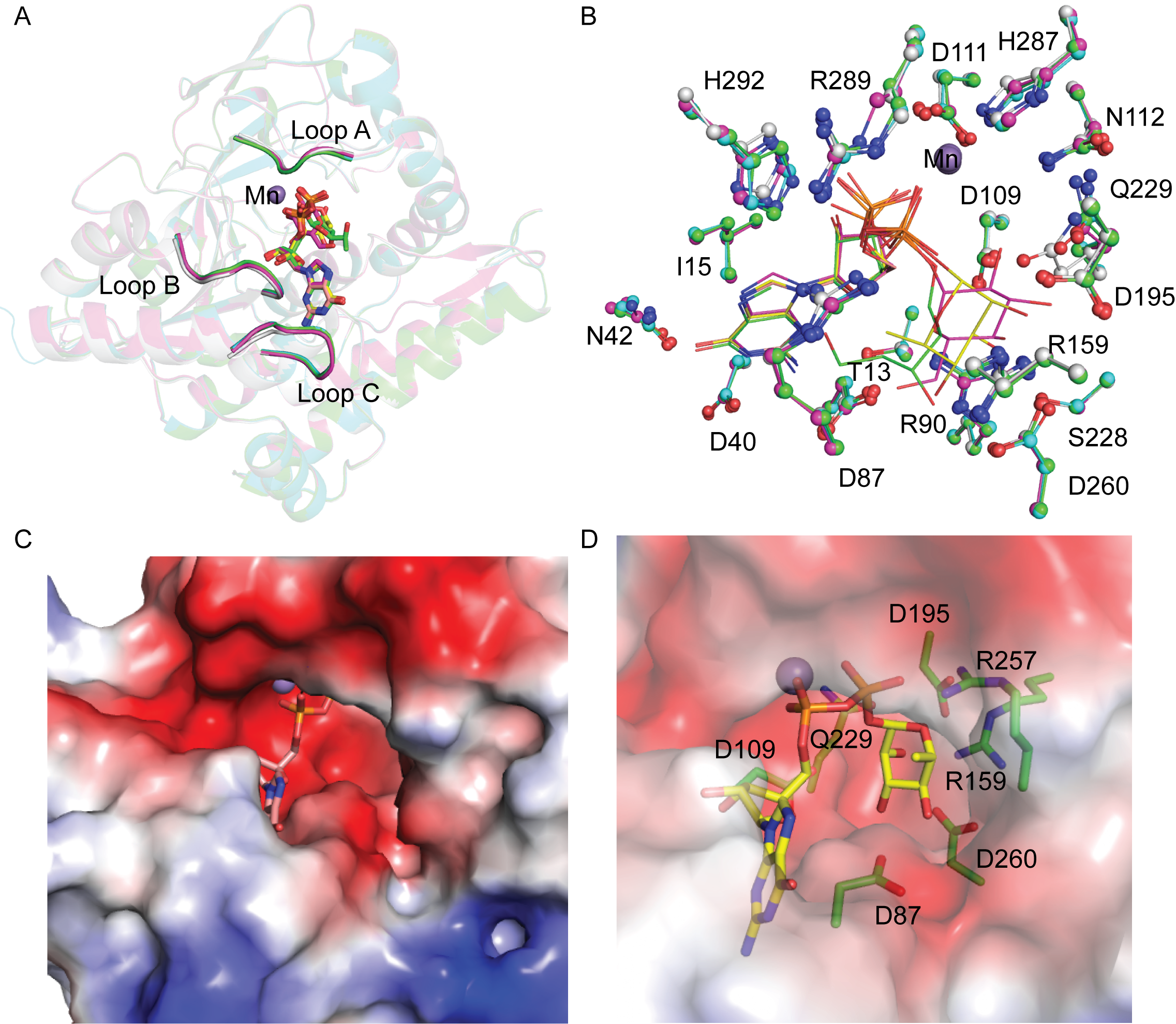


**Fig. S7.** (A) Structural comparison of MtdL·Mn^2+^·GDP, MtdL·Mn^2+^·GDP-D-Glcp, MtdL-S228A·Mn^2+^·GDP-L-Fucp and MtdL-S228A·Mn^2+^·GDP-L-Fucf. The protein was colored gray, cyan, green and magenta, respectively, in the four crystal structures. The D-Galp, L-Fucp and L-Fucf moieties were colored magenta, yellow and green, respectively. (B) Superposition of the substrate binding residues in MtdL·Mn^2+^·GDP, MtdL·Mn^2+^·GDP-D-Glcp, MtdL-S228A·Mn^2+^·GDP-L-Fucp and MtdL-S228A·Mn^2+^·GDP-L-Fucf structures. (C) Electrostatic surface of MtdL·Mn^2+^·GDP structure. (D) Electrostatic surface of MtdL·Mn^2+^·GDP-L-Fucp structure.


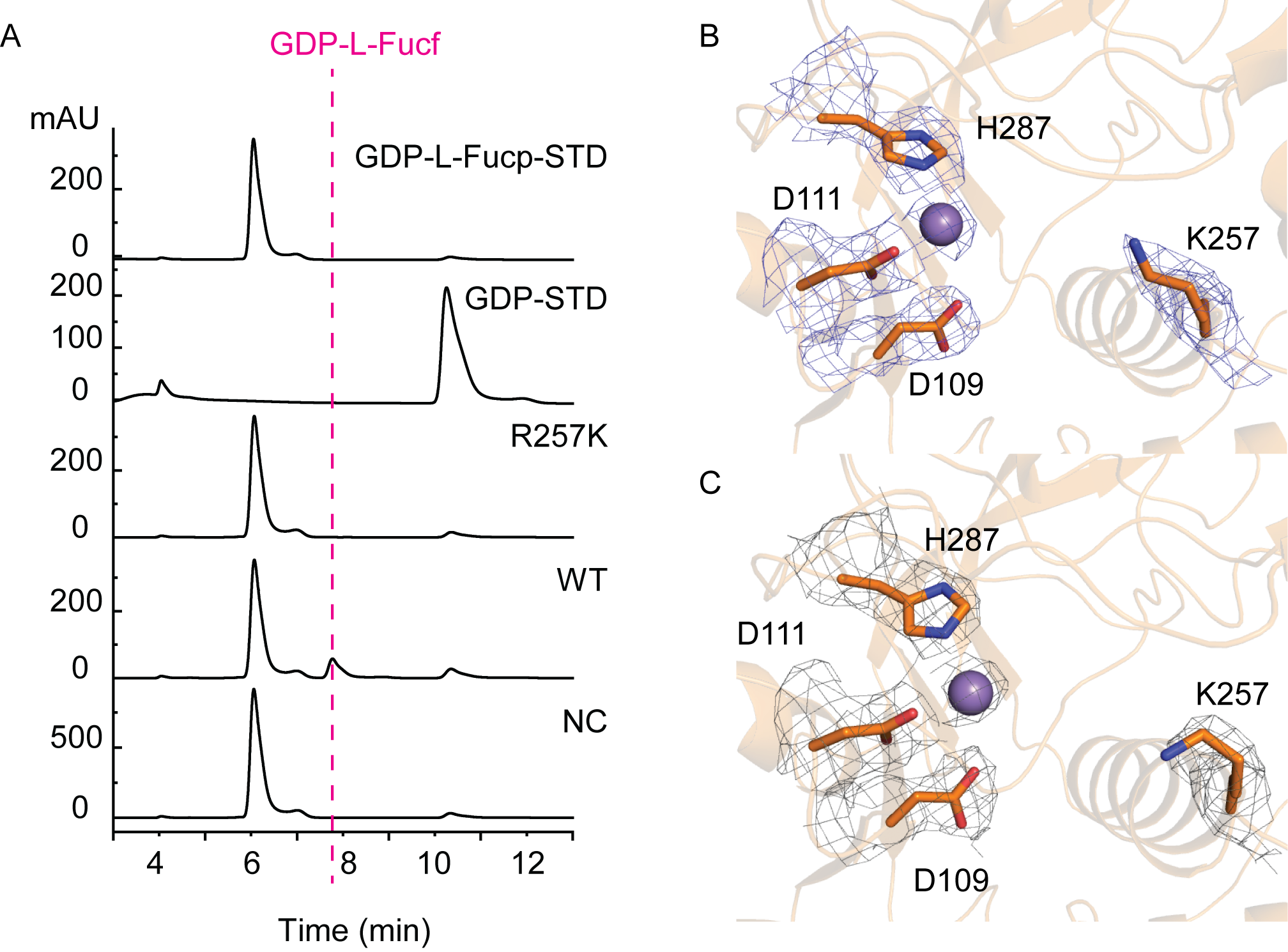
**Fig. S8.** (A) HPLC analyses on a C18 column of the in vitro reaction mixtures catalyzed by MtdL WT and its R257K mutant. (B) 2Fo-Fc electron density maps contoured at 1σ for residues Asp109, Asp111, His287 and Lys257 and contoured at 2σ for the Mn^2+^ ion in the structure of MtdL-R257K Mn^2+^ cocrystallized with GDP-D-Glc. (C) mFo-DFc omit electron density maps contoured at 1σ for residues Asp109, Asp111, His287 and Lys257 and contoured at 3σ for the Mn^2+^ ion in the structure of MtdL-R257K Mn^2+^ cocrystallized with GDP-D-Glc.


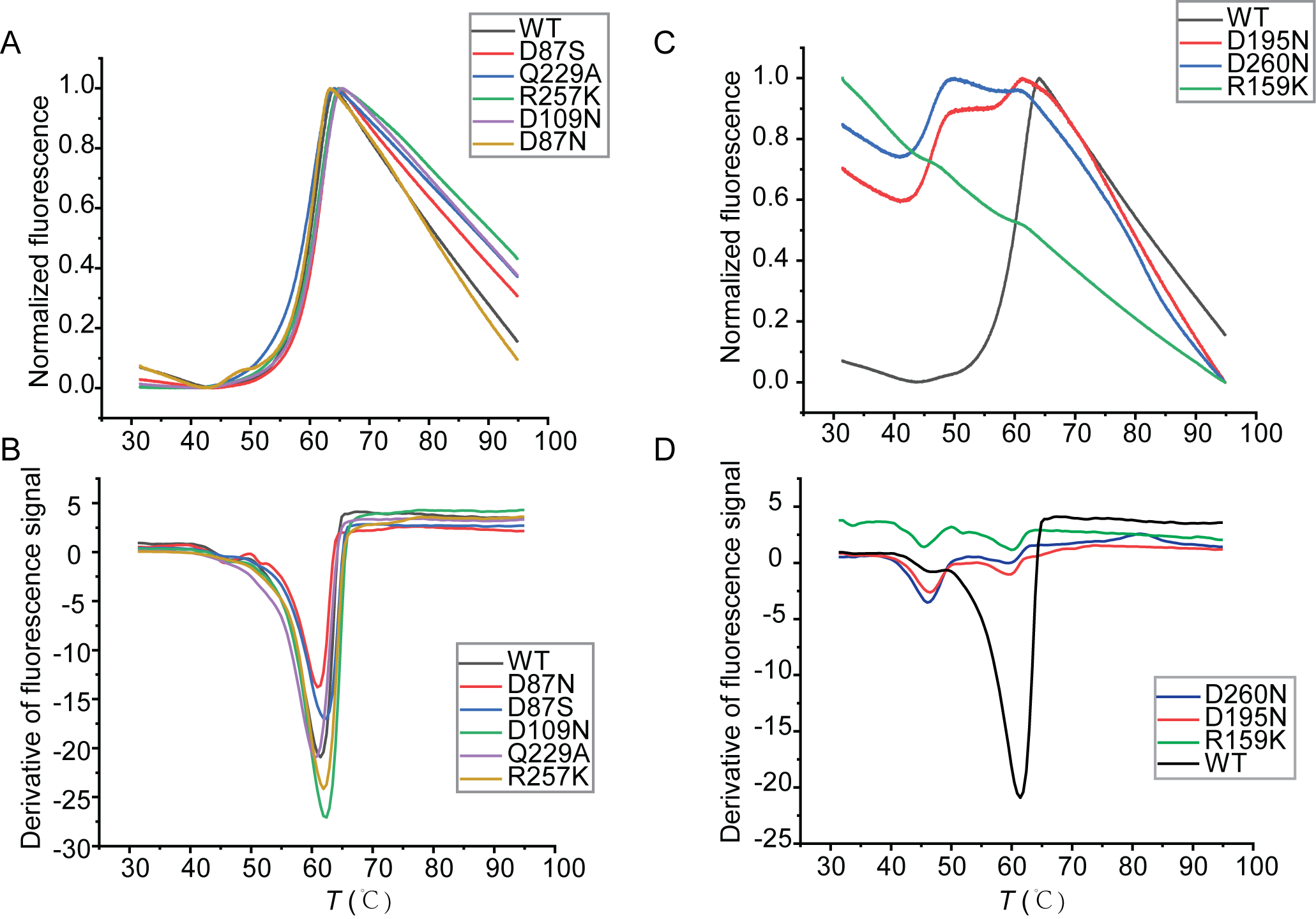
**Fig. S9.** (A) Melting curves of MtdL WT and its D87S, D87N, D109N, Q229A and R257K mutants. (B) The first derivatives of the melting curves of MtdL WT and its D87S, D87N, D109N, Q229A and R257K mutants. (C) Melting curves of MtdL WT and its R159K, D195N and D260N mutants. (B) The first derivatives of the melting curves of MtdL WT and its R159K, D195N and D260N mutants.


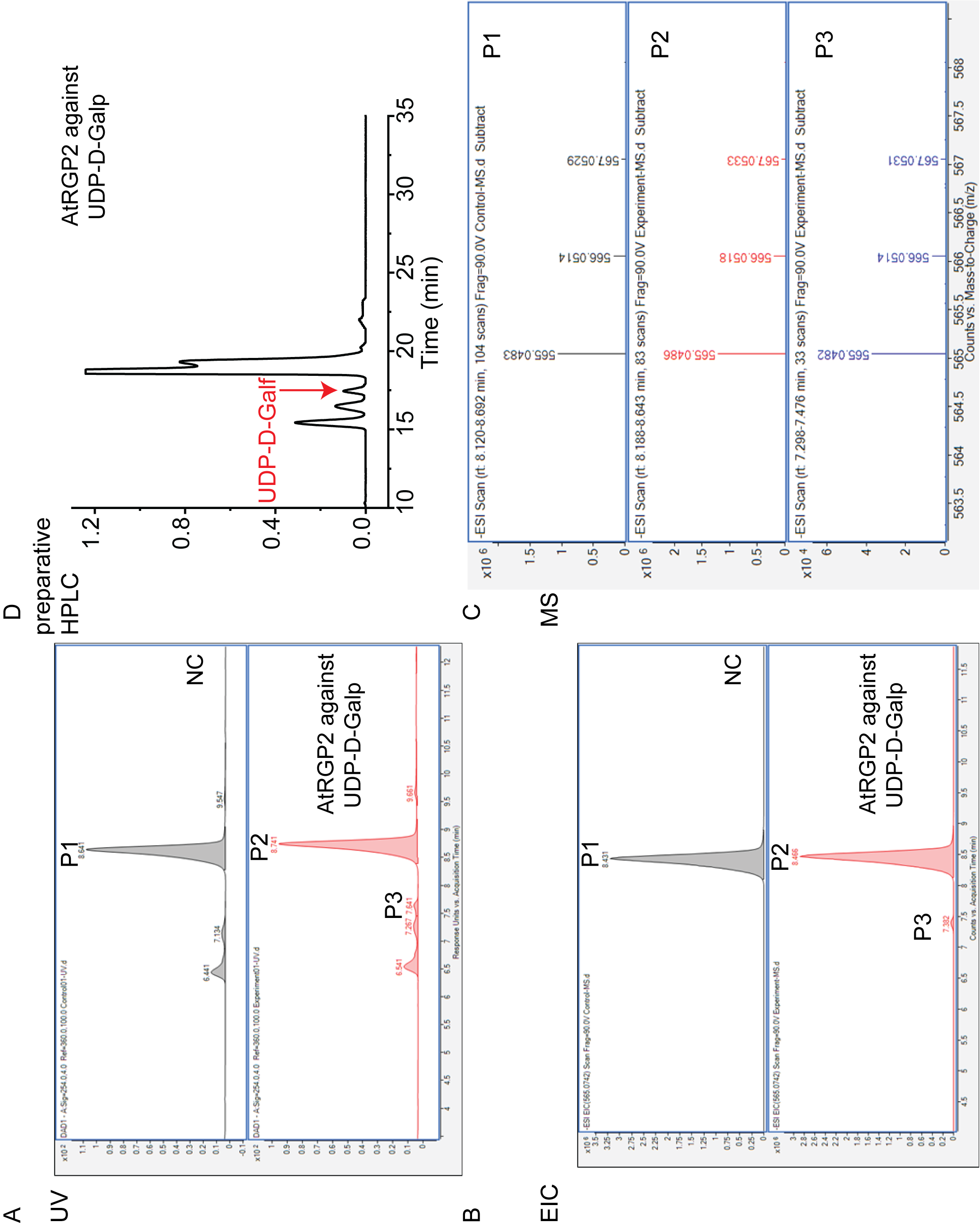


**Fig. S10.** UPLC analyses equipped with a HILIC amide column (A) and EIC-MS analyses (B and C) of AtRGP2-catalyzed reaction mixture in vitro using UDP-D-Galp as substrate. (A) The above chromatogram labeled “NC” represents a negative control which replaced the AtRGP2 enzyme with its dissolved buffer. The below chromatogram labeled “AtRGP2 against UDP-D-Galp” represents the reaction catalyzed by AtRGP2 using UDP-D-Galp as substrate. Peak P1 represents the substrate UDP-D-Galp in the negative control. Peaks P2 and P3 represent the remaining substrate and new enzymatic product in the AtRGP2-catalyzed reaction by comparison to the negative control above. (B) are extracted ion chromatograms of (A) at chosen mass-to-charge value. The delay time of ultraviolet (UV) detector and mass spectrometry detector here was about 0.2 min. (C) The above, middle and below mass spectra correspond to the samples in peaks P1, P2 and P3, respectively. The results indicated that the new enzymatic product corresponding to peak P3 ([M - H]^-^ m/z = 565.0482) had a mass almost identical to that of the substrate UDP-D-Galp ([M - H]^-^ m/z = 565.30). ( D) HPLC purification of the product UDP-D-Galf of AtRGP2 catalysis using a preparative amide column.


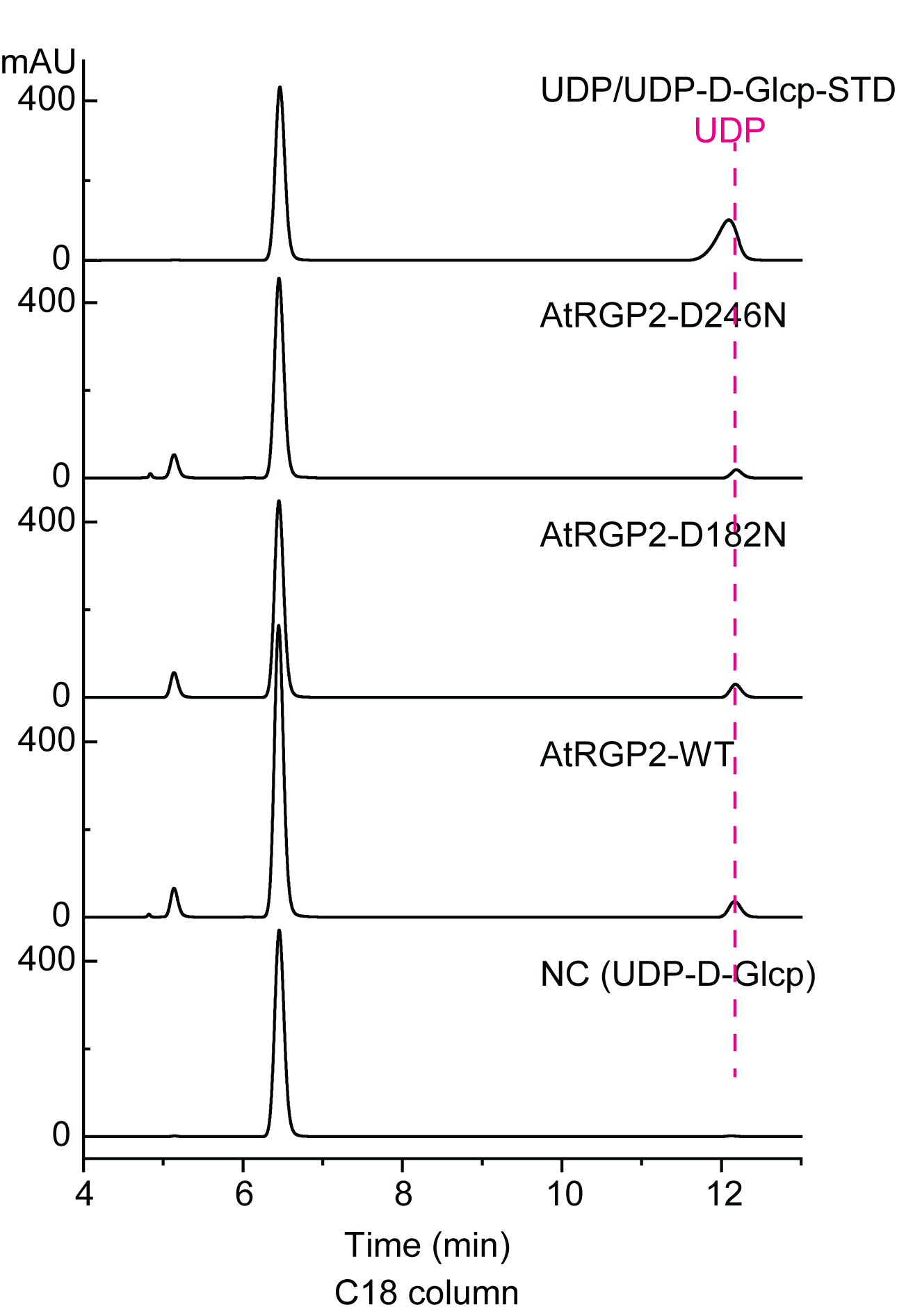


**Fig. S11.** HPLC analyses equipped with a C18 column of the reaction mixtures catalyzed by AtRGP2 WT, D182N and D246N mutants using UDP-D-Glcp as substrate.


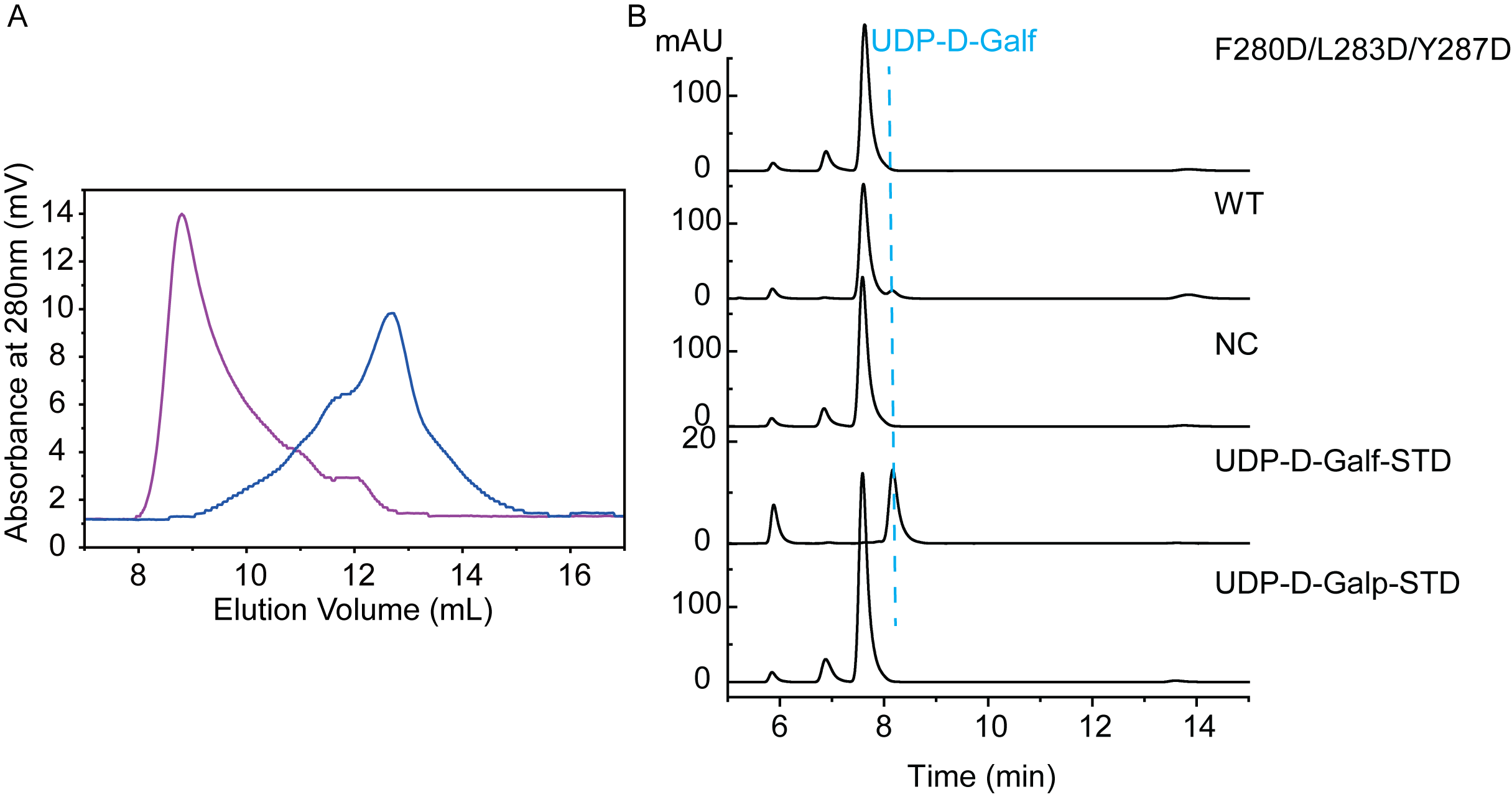


**Fig. S12.** (A) Size exclusion chromatography profiles of recombinant AtRGP2 WT and its F280D/L283D/Y287D mutant shown as purple and blue lines, respectively, with a superdex 200 increase 10/300 GL column after being purified on a nickel column and a superdex 200 HiLoad 16/60 column. (B) HPLC analyses of the in vitro reaction mixtures catalyzed by AtRGP2 WT and its F280D/L283D/Y287D mutant.


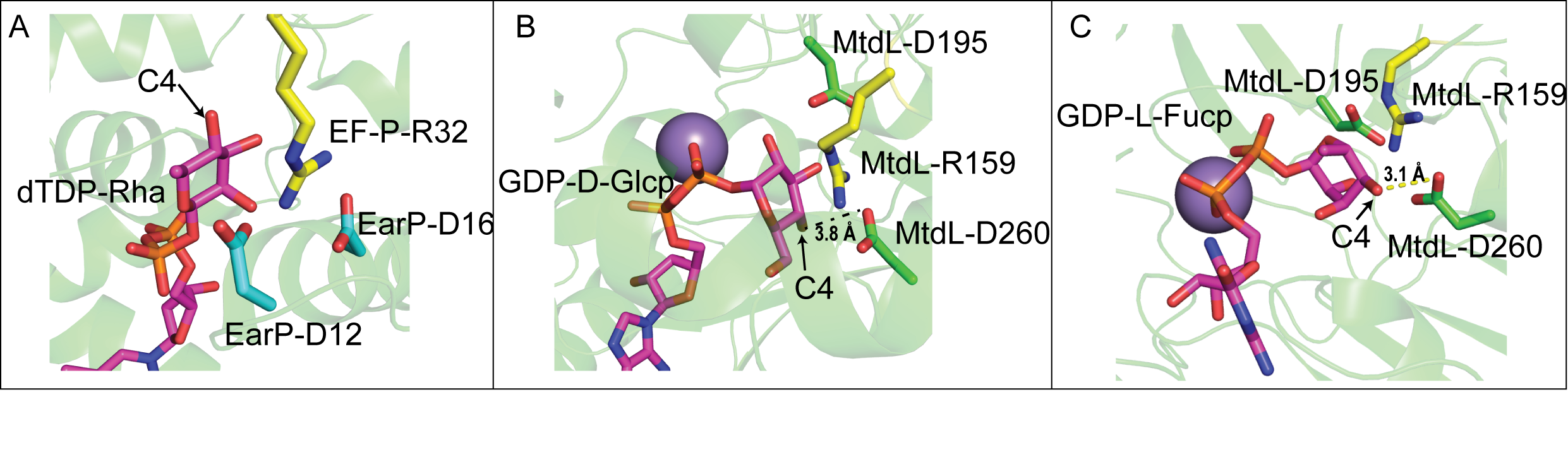


**Fig. S13.** Comparative structural analysis of active sites of two Arginine glycosyltransferases MtdL and EarP. (A) Bacterial EarP specifically transfers rhamnose from dTDP-β-L-rhamnose (TDP-Rha) to Arg32 of translation elongation factor P (EF-P) to activate its function. Structure superposition of EarP active site in complex with TDP-Rha and in complex with TDP and EF-P. (B) Structure of the active site of MtdL in complex with GDP-D-Glcp. (C) Structure of the active site of MtdL in complex with GDP-L-Fucp. Asp260 of MtdL is located in a postion equivalent to that of the catalytic base Asp16 of EarP relative to their respective acceptor arginine residues. In the GDP-L-Fucp complexed structure of MtdL, the C4-OH of L-Fucp is located at a distance of 3.1 Å to the carboxyl group of putative catalytic base Asp260. In the GDP-D-Glcp complexed structure of MtdL, the C4-OH of D-Glcp is located at a distance of 3.8 Å to the carboxyl group of Asp260. By contrast, the C4-OH of L-Rha is located far away from catalytic base Asp16 of EarP.


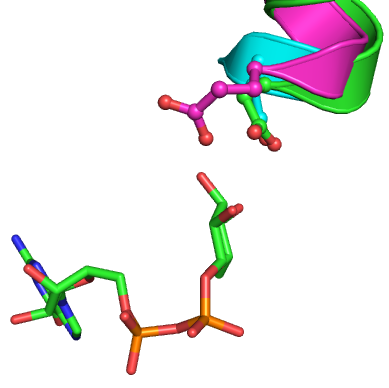


**Fig. S14.** Comparison of the predicted catalytic base in the active site of MtdL (green) in complex with GDP-L-Fucp to those of inverting GnT Ⅰ (cyan, PDB code 2am3) and retaining MAP2569c (purple, PDB code 3ckq). The side chains of the predicted catalytic bases in these structures (Asp260 in MtdL, Glu237 in MAP2569c and Asp291 in GnT Ⅰ) are shown in ball and stick. In MtdL and the inverting GnT Ⅰ, the side chain is on the nucleoside-distal side of the sugar moiety. In the retaining MAP2569c, the side chain is on the nucleoside-proximal side of the sugar moiety.

**Table S1**. Data collection and refinement statistics.

| Crystal | GDP·SeMet-MtdL | | GDP·Mn^2+^·  MtdL | | | GDP-D-Glcp·Mn^2+^·MtdL | | | GDP-L-Fucp·Mn^2+^·MtdL-S228A | | GDP-L-Fucf·Mn^2+^·MtdL-  S228A | Mn^2+^·MtdL-  R257K | |
| --- | --- | --- | --- | --- | --- | --- | --- | --- | --- | --- | --- | --- | --- |
| Wavelength (Å) | 0.9792 | | 0.9792 | | | 0.9792 | | | 0.9792 | | 0.9792 | | 0.9785 |
| Space group | *C*2 | | *C*222_1_ | | | *C*2 | | | *C*222_1_ | | *C*222_1_ | | *C*222_1_ |
| Resolution (Å) | 50.00-2.10 (2.21-2.10) | | 50.00-2.10 (2.16-2.10) | | | 50.00-2.00 (2.05-2.00) | | | 50.00-2.20 (2.27-2.20) | | 50.00-2.40 (2.49-2.40) | | 50.00-2.50  (2.60-2.50) |
| Cell parameters | | | | | | | | | | | | |  |
| a, b, c (Å) | 162.21, 97.48,  57.92 | | 98.69, 162.24, 116.50 | | | 162.28, 98.83,  58.10 | | | 98.19, 162.55, 115.66 | | 98.14,  162.67, 114.93 | | 98.61,  162.40,  116.21 |
| α, β, γ (°) | 90,  97.32,  90 | | 90,  90,  90 | | | 90,  96.77,  90. | | | 90,  90,  90 | | 90,  90,  90 | | 90,  90,  90 |
| *R*_merge_ (%) | 11.2 (82.5) | | 13.4 (93.6) | | | 9.7 (91.9) | | | 15.8 (99.0) | | 15.7 (138.7) | | 17.3(120.7) |
| *CC*_1/2_ | 0.997 (0.822) | | 0.999 (0.939) | | | 0.998 (0.879) | | | 0.998 (0.916) | | 0.999  (0.9) | | 0.997  (0.866) |
| *CC*ano | 0.176 | | - | | | - | | | - | | - | | - |
| *I*/σ*I* | 10.8 (2.2) | | 15.2 (2.8) | | | 11.1 (1.7) | | | 9.9 (2.3) | | 12.1 (2.2) | | 7.8(1.6) |
| Completeness (%) | 98.7 (98.2) | | 99.5 (99.9) | | | 90.2 (93.7) | | | 99.3 (99.2) | | 99.9 (99.9) | | 99.6(99.9) |
| Avg redundancy | 6.9 (6.5) | | 13.3 (13.7) | | | 6.4 (6.3) | | | 9.3 (9.2) | | 13.2 (13.7) | | 7.1(7.3) |
| **Refinement** |  |  | | |  | | |  | | |  | |  |
| No. of reflections overall/test set | 51404  /2682 | | | 54188  /2734 | | | 54968  /2756 | | | 46660  /2174 | 36089  /1823 | | 32243  /1567 |
| *R*_work_/*R*_free_ (%) | 21.06/24.29 | | 22.35/26.74 | | | 21.52/25.90 | | | 23.76/27.98 | | 25.76/29.28 | | 25.65/29.56 |
| No. of atoms |  | | |  | | |  | | |  |  | |  |
| Protein | 5806 | | | 5875 | | | 5849 | | | 5788 | 5776 | | 5777 |
| Ligand | 56 | | | 56 | | | 78 | | | 76 | 76 | | / |
| Mn^2+^ | / | | | 2 | | | 2 | | | 2 | 2 | | 2 |
| H_2_O | 418 | | | 649 | | | 511 | | | 614 | 230 | | 131 |
| B factors (Å^2^) |  | | |  | | |  | | |  |  | |  |
| Protein | 41.18 | | | 32.38 | | | 40.76 | | | 31.02 | 46.14 | | 47.67 |
| Ligand | 68.32 | | | 57.27 | | | 40.53 | | | 30.42 | 48.80 | | / |
| Mn^2+^ | / | | | 62.58 | | | 41.93 | | | 22.12 | 52.81 | | 80.80 |
| H_2_O | 40.64 | | | 39.31 | | | 44.07 | | | 37.05 | 45.03 | | 39.58 |
| RMSD |  | | |  | | |  | | |  |  | |  |
| Bond lengths (Å) | 0.002 | | | 0.002 | | | 0.002 | | | 0.002 | 0.002 | | 0.002 |
| Bond angles (°) | 0.471 | | | 0.553 | | | 0.554 | | | 0.532 | 0.543 | | 0.437 |
| Ramachandran plot residues % | | | | | | | | | | | | |  |
| Favored | 98.78 | | | 98.78 | | | 99.06 | | | 98.64 | 98.1 | | 97.7 |
| Allowed | 1.22 | | | 1.22 | | | 0.94 | | | 1.36 | 1.9 | | 2.3 |

**Table S2**. Melting temperature (*T*_m_) values of MtdL WT and various variants.

| protein | *T*_m_ (℃) |
| --- | --- |
| WT | 60.9±0.03 |
| D87S | 61.7±0.16 |
| D87N | 60.6±0.03 |
| D109N | 61.9±0.08 |
| Q229A | 60.3±0.03 |
| R257K | 61.6±0.02 |
| R159K | 60.2±0.27 |
| D195N | 59.6±0.27 |
| D260N | 59.4±0.5 |
